# Mesohepatectomy Versus Extended Hemihepatectomies for Centrally Located Liver Tumors: A Meta-Analysis

**DOI:** 10.1038/s41598-017-09535-0

**Published:** 2017-08-24

**Authors:** Jianbo Li, Chengdi Wang, Jiulin Song, Nan Chen, Li Jiang, Jiayin Yang, Lunan Yan

**Affiliations:** 1 Department of Liver Surgery and State Key Laboratory of Biotherapy, West China Hospital of Sichuan University, Chengdu, China; 2 Department of Respiratory and Critical Care Medicine, West China Medical School/West China Hospital, Sichuan University, Chengdu, China; 3 West China School of Medicine/West China Hospital, Sichuan University, Chengdu, China

## Abstract

The comparison of Mesohepatectomy (MH) with conventional extended hemihepatectomies (EH) for patients with centrally located liver tumors (CLLTs) were inconsistent. Our aims were to systemically compare MH with EH and to determine whether MH can achieve a similar clinical outcome as EH through this meta-analysis. PubMed/Medline, EMBASE, Web of Knowledge and Cochrane Library were searched updated to June 11, 2016. Blood loss and operation time favored MH in elder patients (mean difference [MD] for blood loss: −692.82 ml, 95% CI: −976.72 to −408.92 ml, P < 0.001; MD for operation time: −78.75 min, 95% CI: −107.66 to −49.81, P < 0.001). Morbidity rate (29.2%, 95% CI: 24.1 to 34.8%), mortality rate (2.0%, 95% CI: 1.2 to 3.3%) and overall survival (median OS 38.2 m, 95% CI: 34.0 to 42.8 m) of MH were comparable with those of EH. The low liver failure rate favored MH (odds ratio [OR]: 0.29, 95% CI: 0.09 to 0.88, P = 0.03). For MH, bile leakage was the most common surgical complication (MH vs. EH: 13.5% vs. 6.7%, P = 0.016), while for EH, it was wound infection (MH vs. EH: 6.9% vs. 15.7%, P < 0.001). Thus MH might be in general safe and feasible for treating CLLTs with a similar clinical outcome as EH.

## Introduction

Centrally located liver tumors (CLLTs) that involve the central part of the liver (Couinaud’s segments IV, V and VIII ± I) are characterized by adjacency to the inferior vena cava (IVC), hepatic vein, portal vein, and hepatic artery, which is a challenge during surgical treatment. Extended hemihepatectomies (EH) is considered as the conventional approach to achieve curative resection of CLLTs. However, EH which will excise 60–85%^1, 2^ of hepatic parenchyma is associated with higher mortality and morbidity rates, mainly on account of the increased risk of postoperative liver failure^3^.

With the increased cumulative experience and recent improved techniques of liver resection, mesohepatectomy (MH) has become a feasible and important option for CLLTs to preserve remnant liver function, especially in patients with liver cirrhosis^1^. As a parenchyma-sparing liver resection, MH could preserve an extra 20% to 25% of liver in comparision relative to EH^2^.

Nevertheless, MH is a technically demanding procedure and infrequently used in clinical practice. Only a limited number of retrospective studies compared MH with EH^1, 4–9^ and the results were discrepant. The aim of this study was to systemically compare MH with EH and to determine whether MH can achieve a similar clinical outcome as EH or not for patients with CLLTs.

## Results

### Search Results

**Figure 1:**
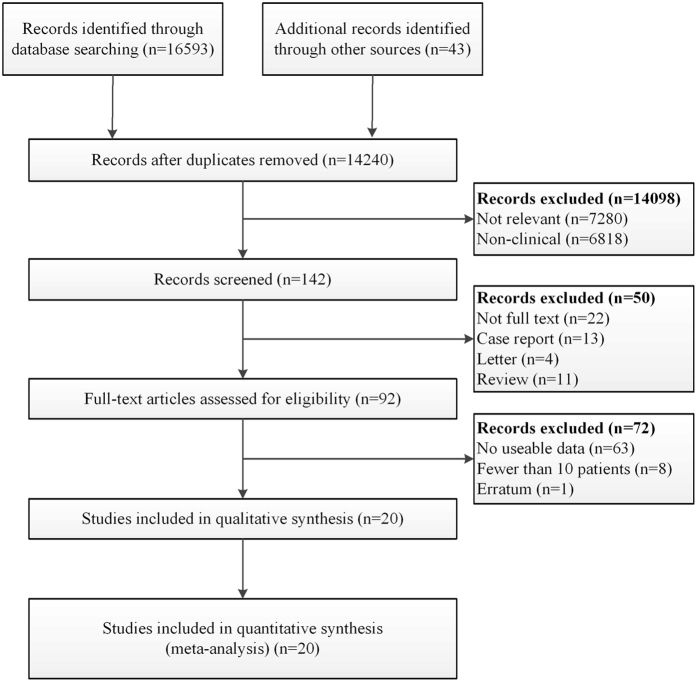
Flow diagram of study identification and selection process.

A total of 16,636 studies were retrieved based on the search strategies described. Ninety-two full-text articles were assessed for eligibility and 72 studies were excluded for no useable data (n = 63), fewer than 10 patients (n = 8), or erratum (n = 1). Finally, 20 studies were selected for evaluation and meta-analysis (Fig. 1), 7 of which reported the results of MH vs. EH^1, 4–9^.

### Characteristics of the Included Studies

The Characteristics of these 20 studies were summarized in Table 1. Overall, The total number of patients was 2,534, of which 1,798 patients enrolled in 20 studies^1, 3–21^ were included in the one arm analysis of perioperative variables and overall survival of CLLTs in patients treated with MH; 908 patients treated with MH and 736 patients treated with EH enrolled in 7 studies were included in the comparison of these two surgical options^1, 4–9^. 11 studies reported the proportion of MH to liver resections directly or indirectly^8, 9, 11–16, 19–21^, and the pooled value showed that this procedure accounted for 4.8% (95% CI: 3.7 to 6.1%) of total liver resections. All studies were retrospective and single-centered. 15 studies were performed in Asia^1, 3–8, 10–13, 15, 18, 19, 21^, 4 in Europe^14, 16, 17, 20^ and 1 in North America^9^.

**Table 1:** Characteristics of the 20 Included Studies.

Ref. Year	Region/Center Description	Type of Operation	n	Sex Man %	Mean/Median	Age Range	The Proportion of MH to Liver Resections (%)	Study Design	Duration of Follow-up (Median/Mean) (Range) (mon)
Zuo^3^	Asia (Mainland China), one center	MH	24	79.17	53.0	27–74	NA	Retrospective	NA
Mehrabi^14^	Europe (Germany), one center	MH	48	60.42	60.7	35–78	5.8 (48/830)	Retrospective	38, 2–72
Chouillard^20^	Europe (France), one center	MH	16	NA	NA	NA	3.7 (16/435)	Retrospective	NA
Lee^19^	Asia (Korea), one center	MH	27	81.48	55.0	28–67	6.2 (27/436)	Retrospective	19.1, 1.4–102.2
Giuliante^16^	Europe (Italy), one center	MH	18	55.56	64.0	53–78	2.5% (18/725)	Retrospective	2–78
			58		41.5	21–75			
Chen^12^	Asia (Mainland China), one center	MH		88.14			5.0 (118/2372)	Retrospective	NA
			60		39.7	18–67			
Arkadopoulos^17^	Europe (Greece), one center	MH	16	61.11	58.0	43–72	NA	Retrospective	NA
	20		61.0	36–76					
Chen^13^	Asia (Mainland China), one center	MH	256	85.94	44.0	NA	5.1 (256/4985)	Retrospective	NA
Chen^10^	Asia (Mainland China), one center	MH	89	84.15	45.5	NA	NA	Retrospective	NA
			157		48.6	NA			
Dai^15^	Asia (Mainland China), one center	MH	17	88.24	52.0	27–71	6.0 (17/285)	Retrospective	NA
Miao^21^	Asia (Mainland China), one center	MH	47	87.23	43.0	19–76	9.6 (92/960)	Retrospective	31
Hasegawa^18^	Asia (Japan), one center	MH	16	50.00	59.3	32–82	NA	Retrospective	NA
Gallagher^11^	Asia (Mainland China), one center	MH	21	90.48	62.0	38–72	1.5 (21/1406)	Retrospective	NA
Wu^8^	Asia (Taiwan China), one center	MH	15	100.00	53.0	32–72	4.1 (15/364)	Retrospective	NA
		EH	25	NA	NA	NA	—		
Scudamore^9^	North America (Canada), one center	MH	18	NA	66.0	NA	7.4 (18/244)	Retrospective	NA
		EH	43	NA	60.0	NA	—		
Qiu^1^	Asia (Mainland China), one center	MH	292	76.71	53.0	NA	NA	Retrospective	NA
		EH	138	78.26	50.0	NA	—		
		MH	118	81.36	56.4	27–78	NA		
Chen^6^	Asia (Mainland China), one center		47		54.9	38–76		Prospective	36.5, 1–96
		EH		83.75			—		
			33		54.0	36–77			
			158		49.2	NA			
Yang^7^	Asia (Mainland China), one center	MH	192	85.14	48.1	NA	NA	Retrospective	NA
		EH	346	83.53	47.5	NA	—		
Hu^4^	Asia (Taiwan China), one center	MH	52	NA	NA	NA	NA	Retrospective	NA
		EH	63	NA	NA	NA	—		
Cheng^5^	Asia (Taiwan China), one center	MH	63	79.37	58.0	NA	NA	Retrospective	30.6, 1.2–99.7
		EH	41	78.05	61.0	NA	—		

MH = mesohepatectomy; EH = extended- hemihepatectomy; 95% CI = 95% confidence interval; mon = month; NA = not available.

### Quality of the Included Studies

Table 2 showed the quality assessment of the 7 comparative studies^1, 4–9^ using the Newcastle-Ottawa Scale (NOS) tool. The NOS is a “star system” in which a study is judged on three broad perspectives: the selection of the study groups, the comparability of the groups and the ascertainment of either the exposure or outcome of interest for case-control or cohort studies respectively. A study can be awarded a maximum of one star for each numbered item within the Selection and Outcome categories. A maximum of two stars can be given for Comparability. There are 4 items in Selection, 1 in Comparability and 3 in Outcome. A study that acquired more than five stars is considered good enough quality study to be included. According to the NOS tool results, the methodological quality of the 7 comparative studies were satisfactory.

**Table 2:** The Quality Assessment for the Seven Studies Involving Nonrandomized Comparison of MH and EH through the Newcastle-Ottawa Scale (NOS).

Ref. Year	NOS Quality Assessment			
	Selection	Comparability	Exposure	Total
Wu^8^	3	2	2	7
Scudamore^9^	3	2	1	6
Qiu^1^	3	2	1	6
Chen^6^	4	2	3	9
Yang^7^	3	2	2	7
Hu^4^	3	2	2	7
Cheng^5^	3	2	3	8
Average	3.143	2	2	7.143

### Baseline Features for MH and EH

To evaluate the two different surgical procedures as a whole, 63 parameters were involved in our data extraction and one arm analysis were used to pool the values (Supplementary Tables S1–S3). Here, we listed the main perioperative variables which included pathogenesis, liver function, tumor markers, morphology, and prognosis (Table 3). Most clinical data of these two procedures were similar. With respect to pathogenesis, most patients had virus infection (85.5% for MH, 78.9% for EH), and a large part of it belonged to the hepatitis B virus (HBV) (73.1% for MH, 78.7% for EH); The proportion of cirrhosis (77.3% for MH, 62.4% for EH) was consistent with that of virus infection. In terms of liver function, most patients were Child-Pugh A (92.4% for MH, 89.5% for EH) and the mean ICG-R15 were 11.4% and 7.9% for MH and EH respectively. For CLLTs, most were solitary (80.6% for MH, 66.6% for EH) and more than half had the tumor capsule (58.2% for MH, 52% for EH); the mean tumor size was about 8 cm (8.6 cm for MH, 8.2 cm for EH). For MH, the operation time were 274 min (95% CI: 236 to 313 min) and average blood loss was 720 ml (95% CI: 578 to 861 ml). However, for EH, the operation time was a little shorter (245 min, 95% CI 219 to 272) and the blood loss was more (1001 ml, 95%CI 743 to 1260) than those in MH. The pooled value also showed that the median OS, 1-, 3 and 5-year overall survival following MH were respectively 38.2 m (95% CI: 34.0 to 42.8 m), 80.8% (95% CI: 76.1 to 85.4%), 54.0% (95% CI: 47.8 to 60.2%) and 42.5% (95% CI: 33.9 to 51.1%), which were similar to EH.

**Table 3:** One Arm Analysis results of Main Perioperative Variables and Overall Survival.

Variables	No. of pooled studies		Mean MH	95% CI	Mean EH	95% CI
	MH	EH				
**Pathogenesis**						
Virus infection (%)	15	5	85.5	71.0–93.5	78.9	51.2–92.9
HBV (%)	12	4	73.1	55.0–85.8	78.7	64.7–88.4
HCV (%)	7	3	11.7	5.0–24.9	17.2	12.4–23.5
Cirrhosis (%)	13	5	77.3	67.3–85.0	62.4	42.9–78.5
**Liver function**						
Total Bilirubin (umol/l)	5	3	15.9	12.4–19.4	17.0	11.8–22.1
Albumin (g/l)	6	3	39.7	37.7–41.6	40.8	39.5–42.0
ALT (U/L)	4	3	47.0	38.0–56.0	50.1	45.4–54.9
PT (s)	3	3	11.6	11.2–11.9	11.7	11.4–11.9
**Child-Pugh Grade (%)**						
A	14	4	92.4	89.2–94.7	89.5	80.2–94.7
B	14	4	7.6	5.3–10.8	10.5	5.3–19.8
ICG-R15 (%)	5	4	11.4	5.7–17.0	7.9	4.0–11.9
**Tumor marker and morphology**						
AFP (ng/ml)	4	—	1991.9	654.6–3329.2	—	—
Number of tumors (solitary%)	8	3	80.6	66.6–89.6	66.6	36.9–87.2
Formation of tumor capsule (%)	6	3	58.2	49.1–66.7	52.0	43.3–60.6
Tumor size (cm)	9	4	8.6	7.3–10.0	8.2	7.8–8.5
**Prognosis**						
Operation time (min)	12	6	274.0	236–313	245.0	219–272
Blood loss (ml)	10	5	720.0	578–861	1001.0	743–1260
weight for resected liver (g)	5	2	526.0	179–872	1029.0	138–1920
overall morbidity (%)	20	7	29.2	24.1–34.8	32.9	17.0–54.0
liver failure rate (%)	10	4	2.5	1.5–4.0	6.7	3.9–11.2
Mortality (%)	18	7	2.0	1.2–3.3	2.8	1.3–5.9
Death of liver failure (%)	8	6	52.2	28.6–75.0	71.8	44.5–89.0
median OS (mon)	9	4	38.2	34.0–42.8	37.7	30.4–46.7
1-year OS	10	4	80.8	76.1–85.4	85.8	78.3–93.2
3-year OS	10	4	54.0	47.8–60.2	56.6	44.9–68.3
5-year OS	10	4	42.5	33.9–51.1	46.0	29.7–62.2

MH = mesohepatectomy; EH = extended hemihepatectomy; 95% CI = 95% confidence interval; HBV = hepatitis B virus; HCV = hepatitis C virus; ALT = alanine aminotransferase; PT = prothrombin time; ICG-R15 = indocyanine green retention rate at 15 min; AFP = alpha-fetoprotein; OS = overall survival.

### Outcome comparison between MH and EH

**Figure 2:**
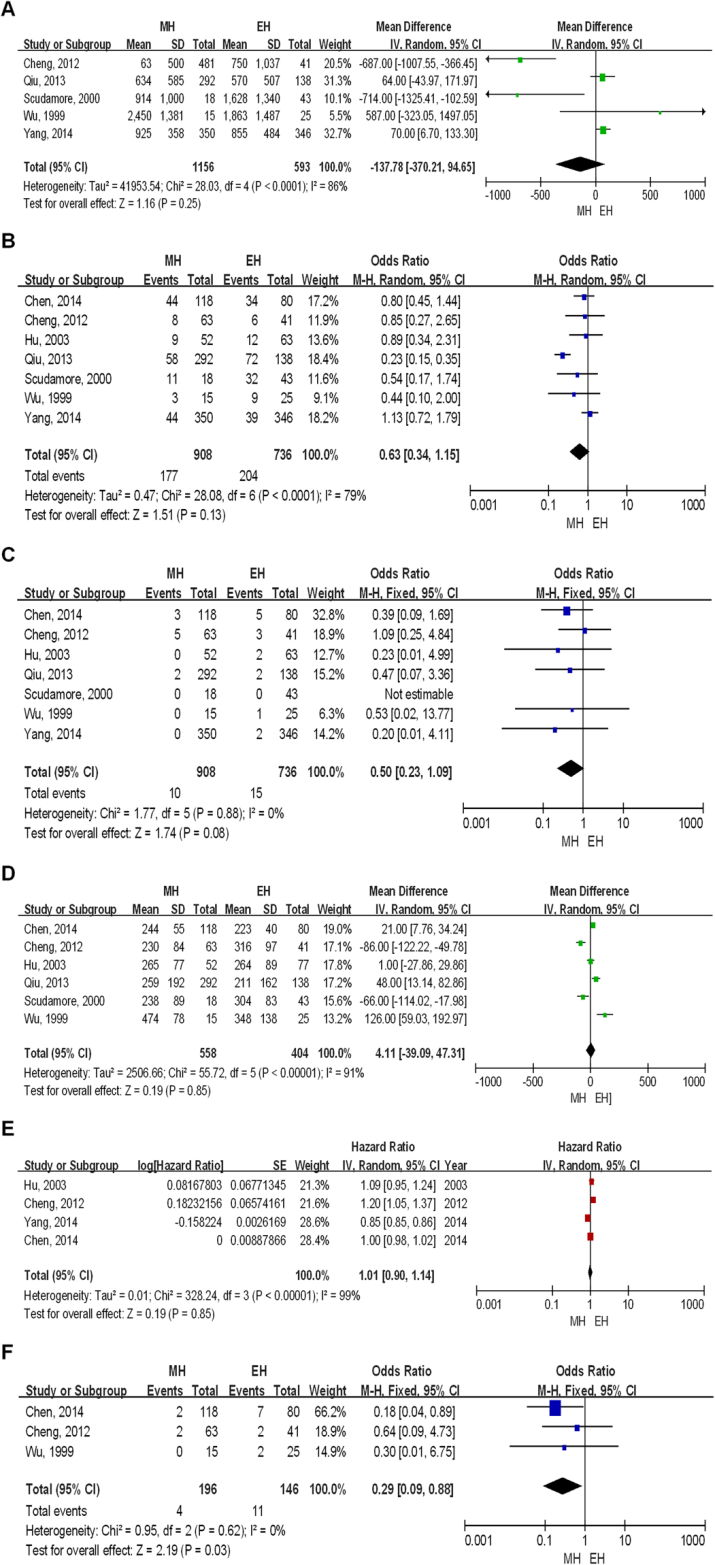
Outcome comparison of the two surgical approaches by two arm analysis. (**A**) Blood loss. (**B**) Morbidity rate. (**C**) Mortality rate. (**D**) Operation time. (**E**) Overall survival. (**F**) Liver failure rate.

To evaluate outcomes of the two surgical approaches by two arm analysis, we selected 6 key prognostic indicators which were blood loss, liver failure rate, morbidity rate, mortality rate, operation time and OS. In these indicators, the pooling of data showed no significant difference in blood loss (Table 4 and Fig. 2A), morbidity rate (Table 4 and Fig. 2B), mortality rate (Table 4 and Fig. 2C), operation time (Table 4 and Fig. 2D) and OS (Table 4 and Fig. 2E). However, results showed MH had significant advantages over EH in reducing liver failure rate (Table 4 and Fig. 2F).

**Table 4:** Two Arm Analysis Results of MH and EH.

Variables	No. of Pooled Studies	Model	Heterogeneity		OR/HR/MD	Meta-analyses	
			*I* ^2^	p		95% CI	p
Blood loss	5	random	86%	<0.10	−137.78^‡^	−370.21–94.65	0.25
Morbidity rate	7	random	79%	<0.10	0.63^†^	0.34–1.15	0.13
Mortality rate	7	fixed	0%	0.88	0.50^†^	0.23–1.09	0.08
Operation time	6	random	91%	<0.10	4.11^‡^	−39.09–47.31	0.85
Median OS	4	random	99%	<0.10	1.01^§^	0.90–1.14	0.85
Liver failure rate	3	random	0%	0.62	0.29^†^	0.09–0.88	0.03

MH = mesohepatectomy; EH = extended hemihepatectomy; OR = odds ratio; HR = hazard ratio; MD = mean difference; *I* ^2^ = the percentage of total variation across studies that is due to heterogeneity rather than chance; 95% CI = 95% confidence interval; OS = overall survival; ^†^OR; ^§^HR; ^‡^MD.

When we explored the sources of heterogeneity, subgroup analysis based on patient characteristic of mean age indicated MH was superior to EH regarding blood loss (Supplementary Fig. A) and operation time (Supplementary Fig. D1 and B2) in elder patients (>60 years). The subgroup analysis by type of MH (that is, including segment I resection ν not including segment I resection) (Supplementary Table S2) did not suggest apparent differences in blood loss (Supplementary Fig. E), morbidity rate (Supplementary Fig. F), mortality rate (Supplementary Fig. G) and operation time (Supplementary Fig. H). The pooled OS had a high heterogeneity, but failed us to conducted subgroup analysis due to a small number of studies included. We did sensitivity analysis via removing the study manifested by high heterogeneity, however, this did not changed the pooled effect (Supplementary Fig. I).

### Indications, Complications, Recurrence and Methods of hepatic blood occlusion

**Figure 3:**
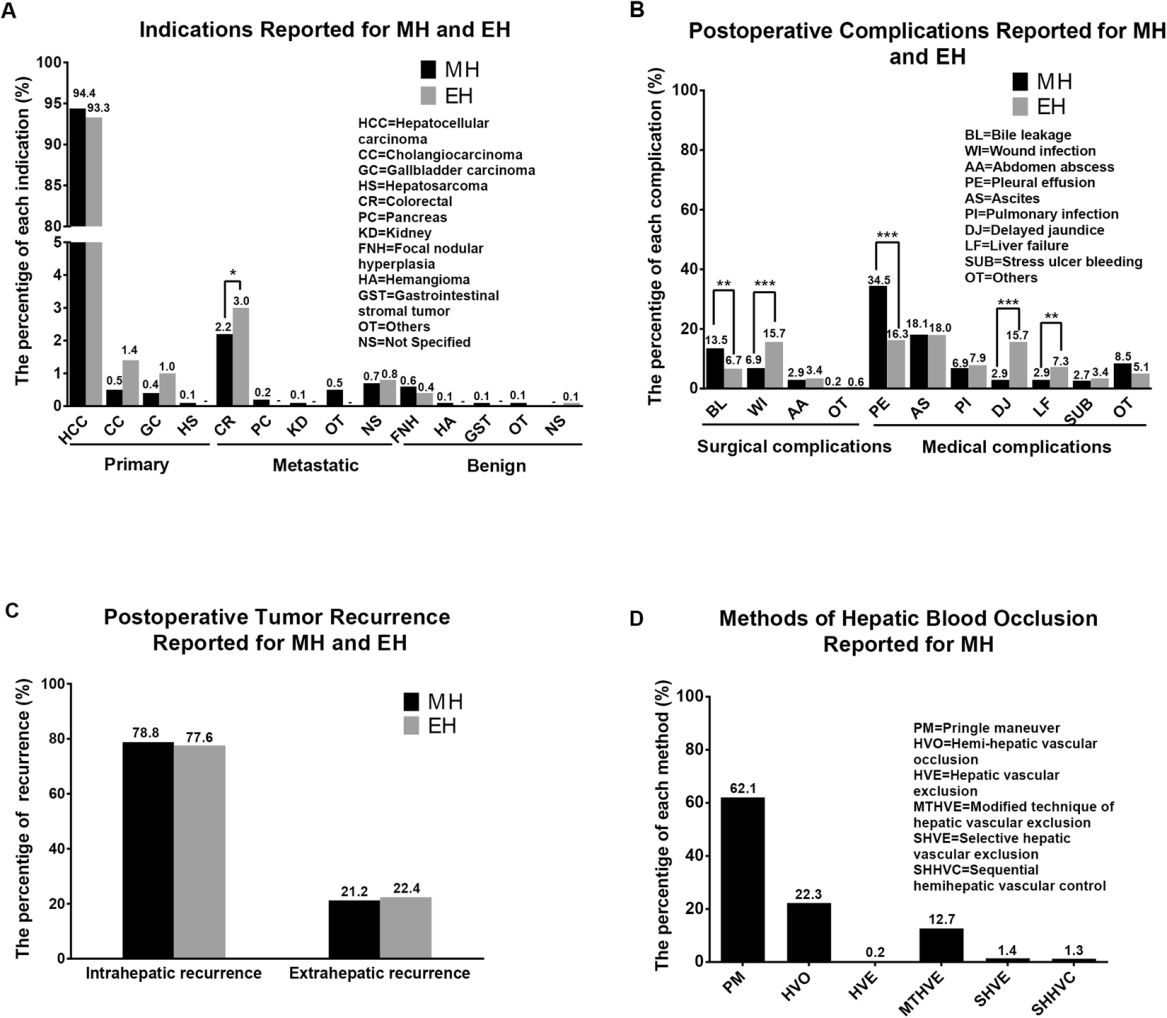
Indications, complications, recurrence and methods of hepatic blood occlusion. (**A**) Indications reported for MH and EH. (**B**) Postoperative complications reported for MH and EH. (**C**) Postoperative tumor recurrence reported for MH and EH. (**D**) Methods of hepatic blood occlusion Reported for MH.

In Fig. 3 and Supplementary Table S4–S7, we summarized the indications, complications, recurrence and methods of hepatic blood occlusion reported for MH and/or EH. For MH, there was a total of 1701 patients diagnosed with primary malignant diseases of liver, out of a total of 1782 patients (Supplementary Table S4). Hepatocellular carcinoma (HCC) was the most common diagnosis, which accounted for 94.4% of all indications. In liver metastatic tumor, the metastasis from colorectal tumor was the most commonly happened, given a frequency of 2.2%. The liver benign diseases had the least frequency of 0.8%. A similar constituent frequency of indications occurred in EH with the exception of a little difference in cholangiocarcinoma (p = 0.024) (Fig. 3A).

Data on complications following MH were available in all studies included, with reported overall morbidity rates ranging from 12.7% to 61.1% (Supplementary Table S3) and a pooled overall morbidity rate of 29.2% (95% CI: 24.1–34.8%) (Table 3). To get the type of postoperative complications reported for MH and EH to the bottom, a detailed systemic table was made (Supplementary Table S5 and Fig. 3B). Bile leakage was the most commonly reported surgical complication following MH, which accounted for 13.5% (65 events) of all complications (MH vs. EH: 13.5% vs. 6.7%, P = 0.016). However, the most commonly reported surgical complication following EH was wound infection, which accounted for 15.7% (28 events) of all complications (MH vs. EH: 6.9% vs. 15.7%, P = 0.000). In medical complications, it deserves to be specially noted that the proportions of delayed jaundice and liver failure following MH were significantly smaller than those following EH (Delayed jaundice following MH vs. EH: 2.9% vs. 15.7%, P = 0.000; Liver failure following MH vs. EH: 2.9% vs. 7.3%, P = 0.012).

We also went into postoperative tumor recurrence seriously and put the analytic results into Supplementary Table S6 and Fig. 3C. There was a total of 585 patients treated with MH had intrahepatic recurrence, out of a total of 742 patients, which gave a frequency of 78.8%. Extrahepatic recurrence following MH occurred in 157 patients, accounted for 21.2%. 194 patients out of a total number of 250 had intrahepatic recurrence following EH, with a proportion of 77.6%, and the other 22.4% patients had extrahepatic recurrence. In consideration of a large percentage made up by “Not Specified”, we gave up to compare the subsections of recurrence between MH and EH to avoid inappropriate results.

In Supplementary Table S7 and Fig. 3D, we summarized the methods of hepatic blood occlusion reported for MH. In total, there were six different hepatic blood occlusion methods for MH. They were Pringle maneuver^1, 3, 5, 7–10, 12–14, 16, 21^, Hemi-hepatic vascular occlusion^5–9, 11, 15, 18–21^, Hepatic vascular exclusion (HVE)^3^, Modified technique of hepatic vascular exclusion (MTHVE)^12, 13^, Selective hepatic vascular exclusion (SHVE)^16, 17^, and Sequential hemihepatic vascular control (SHHVC)^17^. Except for SHHVC, the left 5 occlusion methods were nearly 100% intermittent. The Pringle maneuver and Hemihepatic vascular occlusion were classical occlusion methods for MH, accounted for 62.1% and 22.3% of all methods, respectively. The HVE was seldom used, only accounted for 0.2%. The MTHVE, SHVE and SHHVC were modified with orthodox occlusion methods and reported in 2006^12^, 2008^16^ and 2012^17^, respectively.

### The Relationship between Weight for Resected Liver and Liver Failure Rate

**Figure 4:**
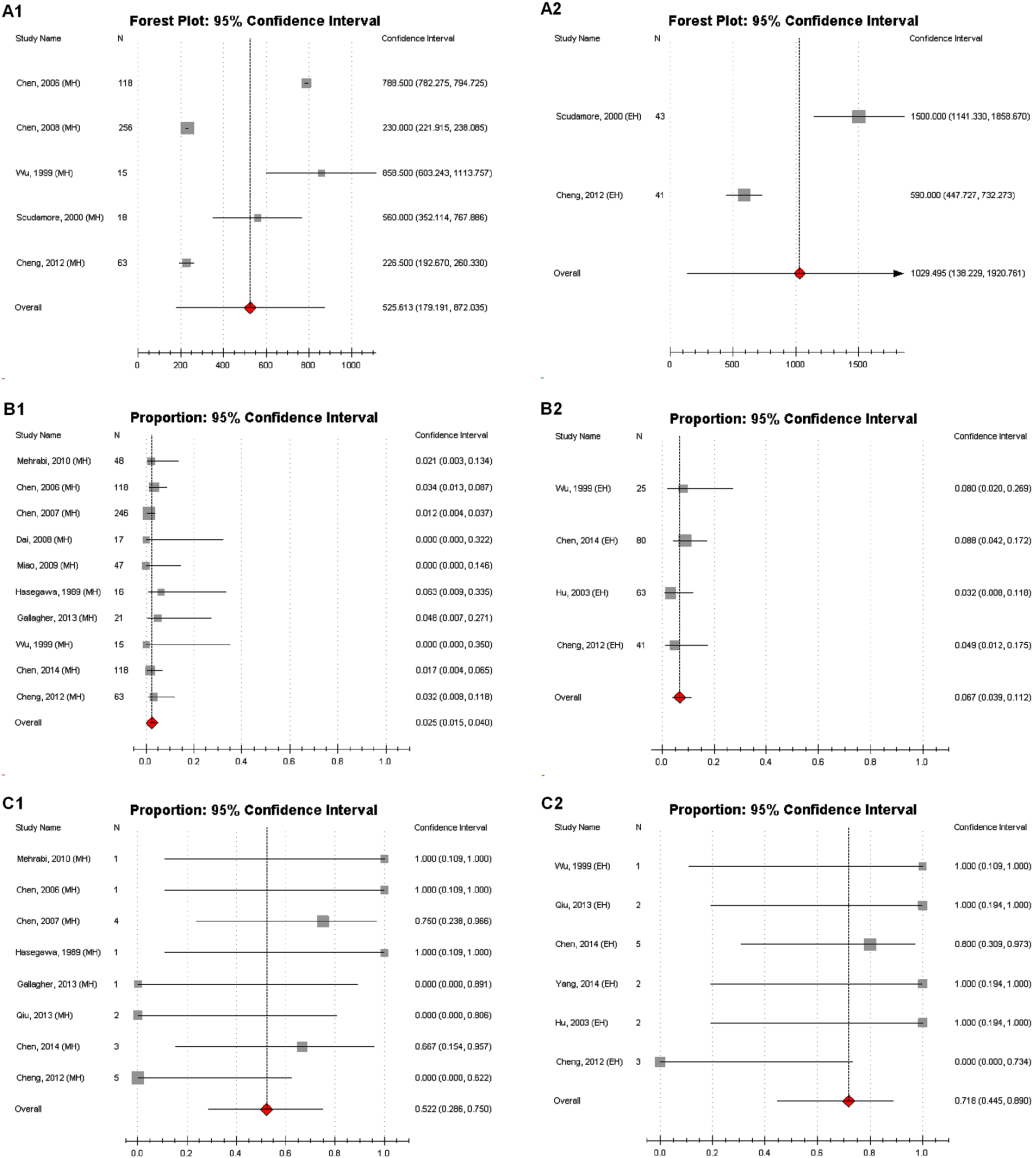
One arm analysis of MH and EH. (**A1**) Mean weight for resected liver in MH. (**A2**) Mean weight for resected liver in EH. (**B1**) Liver failure rate for MH. (**B2**) Liver failure rate for EH. (**C1**) Die of liver failure following MH. (**C2**) Die of liver failure following EH.

The greatest strength of MH was to spare hepatic parenchyma in order to preserve liver function. In this study, we specifically explored the relationship between weight for resected liver and liver failure rate. In our stuy, data of mean weight for resected liver in MH which ranged from 227 g to 859 g and was pooled to be 526 g (95% CI: 179–872 g) (Table 3, Fig. 4-A1 and Supplementary Table S2). However, this pooled weight increased to 1029 g (95% CI: 138–1920 g)^5, 9^ in EH (Table 3, Fig. 4-A2 and Supplementary Table S2). The pooled value showed a lower liver failure rate in MH than that in EH (MH: 2.5%; EH: 6.7%)^4^ (Table 3, Fig. 4-B1 and B2). Liver failure, uncontrollable bleeding, sepsis and severe pulmonary infection were causes reported for postoperative death (Supplementary Table S3). Of all causes for postoperative death, liver failure was the most common one which accounted for 52.2%^1, 5, 6, 10–12, 14, 18^ following MH and 71.8% following EH^1, 4–8^ (Table 3 and Fig. 4-C1 and C2).

## Discussion

MH was first reported to treat central hepatic tumor by Wu *et al*.^22^ in 1965. Although this operation has existed for more than five decades and performed by more medical institutions in recent years, there was still no universal terminology of this surgical procedure^2, 11, 23–25^. Nearly ten different terms has been used to describe this operation^8, 18, 19, 26–31^ (Supplementary Table S9). To avoid confusion, we preferred to call this operation as mesohepatectomy, which was first strongly recommend by Wu^8^ in 1999. MH was first defined as the removal of the right anterior segment in continuity with the left medial segment by McBride^32^ in 1972 and then defined as en bloc resection of Couinaud’s segments IV, V and VIII^8^ or removal of liver segments drained by the middle hepatic vein^31^. The caudate lobectomy was first added to this operation for advanced gallbladder cancer to obtain a wide resection margin by Ogura *et al*. in 1998^33^. In this review, we defined MH as the resection of Couinaud’s segments IV, V and VIII ± I^1, 3, 5, 6, 9, 10, 13, 17^.

Theoretically, the liver failure is associated with the weight of resected liver and our review verified this viewpoint. The postoperative mortality rates were low both in MH and EH and the pooled OR also showed no significant differences between MH and EH. Consistent with the other three reviews^2, 14, 34^, our review also revealed that liver failure was the most common cause among all causes for postoperative death, which accounted for 52.2% following MH. This ratio however, increased by nearly 20% following EH.

Consistent with Lee’s review^2^, bile leakage was the most commonly reported surgical complication following MH. The possible reason suggested by reports for high incidence of bile leakage in MH are as follows: the division of small intrahepatic duct branches at the 2 wide section planes and another one is the exposure of the superior surface of the common bile duct, which potentially lead to the injury of its small branches^4, 35^. Ishii *et al*.^36^ recommended primary closure of the site of bile leakage and/or placement of biliary drainage tubes in cases involving intraoperative bile leakage.

Wound infection belongs in surgical site infection (SSI), which is the most common postoperative complication for hepatic resection^37^. Yang’s study^37^ investigated the risk factors of SSI, and his results didn’t show a significant difference in incisional SSI when major resection (resection of 3 or more Couinaud liver segments) compared with minor resection (resection of fewer than 3 segments). However, MH and EH are both major resection, and our analysis result did show a higher wound rate in EH. It is certain that whether and how the parenchymal preserving surgery such as MH affect would infection should be addressed in future studies.

Blood loss is regarded as a main goal of liver surgery^14, 16^. In our review, the pooled MD of blood loss favored MH, but not significantly. However, subgroup analysis indicated MH was superior to EH regarding blood loss in elder patients (>60 years), which has not been reported in present articles.

The theoretical longer operation time of MH compared to EH is obvious. The possible causes include requirement of meticulous transection attributed to the important vessels surrounded lesions, dissection of more liver parenchyma because of two wide resection planes and sometimes needing cholangioplasty^6, 11^. In Lee’s review^2^, however, the operative time was not increased for experienced surgeons. In our review, the pooled MD showed that MH was comparable to EH regarding operation time and even shorter in elder patients.

Our systematic review and meta-analysis has some limitations. Firstly, the comparability of our included studies was most likely hampered by the use of different definitions for outcome indicators, such as postoperative liver failure, bile leakage and wound infection. Regarding  postoperative  liver failure, only two studies^6, 14^ pointed out that they adopted the “50-50 criteria”  (prothrombin time < 50% and  serum bilirubin > 50 µmol/L on postoperative day 5) to define this outcome indicator, however, the other studies in our analysis failed to give the definition. A similar situation happened to postoperative bile leakage and wound infection. Our results should be interpreted with caution because of the lack of the statement about the definition in outcome events. Secondly, a high risk was associated with the number of studies included. The small number of studies included in pooling OS thwarted our intention to conduct subgroup analysis. We removed the study with high heterogeneity in the sensitivity analysis for OS, nevertheless, we recognized the flaw of this method because it may increase the chance of bias^38^. In addition, the absence of heterogeneity for pooled liver failure rate may be not the result of homogenous data, but the small number of studies included. Finally, some degrees of publication bias and selection bias potentially exist. We selected only English articles published with full text for meta-analysis and excluded the information only in abstract, non-English articles and text books published.

## Conclusion

Our systemic review and meta-analysis suggests that MH is safe and feasible for treating CLLTs and can achieve a similar clinical outcome as EH. Compared with EH, MH decreased blood loss and shorten operation time in the elder and lowered liver failure and wound infection rates. The morbidity rate, mortality rate, and overall survival do not differ between MH and EH.

## Materials and Methods

### Search Strategy

A systemic search of PubMed/Medline, EMBASE, the Web of Knowledge and the Cochrane Library was performed using the strategy as follows: [“middle hepatic lobectomy” OR “median segmentectomy” OR “middle lobectomy” OR “central bisegmentectomy” OR “central hepatectomy” OR “mesohepatectomy” OR “central liver resection” OR “segmentectomy” OR “bisegmentectomy” OR “central trisegmentectomy” OR “bisectionectomy” OR “segmental liver resection” OR “segment oriented liver resection”] OR [(“liver resection” OR “hepatic resection” OR “hepatectomy” OR “hemihepatectomy”) AND (“centr*” OR “mid*” OR “med*”)]. We limited the language to English and the dates from January 1809 to June 11, 2016. Bibliographies of review articles were also hand-searched to identify potentially relevant articles. Studies were included if they met the following criteria: (1) included more than 10 patients; (2) reported perioperative variables and/or outcomes of MH and/or EH for CLLTs in adult patients; (3) were original complete publications in English with full-text accessible; (4) included the most comprehensive study when multiple studies contained overlapping data on the same subject. Case reports, reviews, letters, and editorials were excluded. Two reviewers independently participated in the initial screening based on the above-mentioned inclusion and exclusion criteria and reviewing of full-text articles and quality assessment. Then, a cross-check was performed to identify discrepancies by the same two reviewers. Any conflicts arising between the two reviewers were adjudicated by a third reviewer. The studies’ identification, inclusion, and exclusion were conducted according to PRISMA^39^ (preferred reporting items for systemic reviews and meta-analyses) guidelines (Fig. 1).

### Data Extraction and quality accessment

The data involving 63 parameters was extracted by two reviewers independently. Divergences were resolved by consensus after discussion. The following information were extracted: author, ethnicity, type of operation, study design, age and gender of subjects, follow-up duration, comparison of outcomes in both groups. The Newcastle-Ottawa Scale (NOS)^40^ (Supplementary Material S1) was used to assess the quality of the studies included in this review.

### Data Synthesis and Analysis

We used Review Manager 5.3 and Meta-Analyst 3.13 to pool data. *I* ^2^ tests were used to assess heterogeneity for each pooled analysis and a P value of <0.10 or *I* ^2^ >50% suggested a considerable heterogeneity. If heterogeneity existed, we used the random-effects model to calculate pooled values, otherwise, used a fixed-effect model. For dichotomous data, we used Mantel-Haenszel methods to calculate pooled odds ratio (OR) and for continuous data, we used Inverse Variance methods to calculate pooled mean difference (MD). For time-to-event data, we used the generic inverse variance method to summarize statistics, expressed in hazard ratio (HR). We explored sources of heterogeneity with two priori subgroup hypotheses: mean age of patients (<60 years *v* > 60years) and type of MH (including segment I resection *v* not including segment I resection) and we required the number of more than or equal to five in our subgroup analysis. All the P values were two tailed, and statistical significances was set as P < 0.05.

## Electronic supplementary material

Supplementary information
