# Supplementary material for: Mesohepatectomy Versus Extended Hemihepatectomies for Centrally Located Liver Tumors: A Meta-Analysis
Source: Sci Rep. 2017 Aug 24;7:9329. doi: 10.1038/s41598-017-09535-0 (PMC5571172; doi:10.1038/s41598-017-09535-0)
Supplement: Supplementary file 1 — Supplementary information [file 41598_2017_9535_MOESM1_ESM.pdf]

Mesohepatectomy Versus Extended Hemihepatectomies for Centrally Located Liver Tumors: A Meta-Analysis

Running title: Mesohepatectomy for Centrally Located Liver Tumors

Jianbo Li[1,&], Chengdi Wang[2,&], Jiulin Song[1], Nan Chen[3], Li Jiang[1], Jiayin Yang[1*], Lunan Yan[1*]

[1] Department of Liver Surgery and State Key Laboratory of Biotherapy, West China Hospital of Sichuan University, Chengdu, China

[2] Department of Respiratory and Critical Care Medicine, West China Medical School/West China Hospital, Sichuan University, Chengdu, China

[3] West China School of Medicine/West China Hospital, Sichuan University, Chengdu, China

*To whom correspondence should be addressed:

Prof. Lunan Yan

Department of Liver Surgery & State Key Laboratory of Biotherapy, West China Hospital of Sichuan University, Chengdu 610041, P.R. China. Tel: +86-28-85422867, Fax: +86-28-85422867,

E-mail Address: yanlunan8060@163.com

Prof. Jiayin Yang

Department of Liver Surgery, West China Hospital, Sichuan University, Chengdu, 610041, P.R. China. Tel: +86-18980602047, Email address: doctoryjy@scu.edu.cn

[&] These authors contributed equally to this work.

Supported by a grant from the National Science and Technology Major Project of China (Grant number 81470037 to Prof. Jiayin Yang)

Supplementary table 1. Extracted data and pooled values of preoperative variables for the 20 included studies

Ref. Year	Type of Operation	Blood Vessel Invasion (%)	Cirrhosis (%)	Preoperative TACE/TACI(%)	Virus Infection (%)	Total Bilirubin (Mean/Median, umol/l)	Albumin (Mean/Median,g/l)	ALT/AST (Mean/Median, U/L)	PLT (Mean/Median, 10^9/l)	PT (Mean/Median, s)	Child-Pugh Grade (%)	ICG-R15 (Mean/Median) (%)	AFP (Mean/Median, ng/ml)	Number of Tumours (Solitary %)	Formation of Tumor Capsule (%)	Tumor Size (Mean/Median, cm) (Range, cm)
Zuo, 2014[3]	MH	NA	100 (24/24)	NA	95.8 (23/24, HBV 19 HCV 2 Both 2)	NA	NA	NA	NA	NA	A: 87.5 (21/24) B: 12.5 (3/24)	NA	NA	NA	41.7 (10/24)	6.3 2.0-14.0
Mehrabi, 2008[14]	MH	NA	NA	NA	NA	NA	NA	NA	NA	NA	NA	NA	NA	NA	NA	NA
Chouillard, 2003[20]	MH	NA	NA	NA	NA	NA	NA	NA	NA	NA	NA	NA	NA	NA	NA	NA
Lee, 2008[19]	MH	NA	100 (27/27)	59.3 (16/27)	85.2 (23/27, HBV 20 HCV 3)	NA	NA	NA	NA	NA	A: 100 (27/27)	9.7	11	NA	NA	4.3 2.0-9·0
Giuliante, 2008[16]	MH	NA	NA	NA	NA	NA	NA	NA	NA	NA	NA	NA	NA	NA	NA	5.2 3.0-12.0

Chen, 2006[12]	MH	NA	100<br>(58/58)	8.6<br>(5/58)	98.3<br>(57/58,<br>HBV 51<br>HCV 6)	17.1	42.0	ALT<br>40.5<br>AST<br>22.5	178.5	NA	A: 90.9<br>(50/55)<br>B: 9.1<br>(5/55)	NA	1135	82.8<br>(48/58)	NA	10.4<br>5.6-19.5
			100<br>(60/60)	6.6<br>(4/60)	96.7<br>(58/60,<br>HBV 54<br>HCV 4)	17.1	41.0	ALT<br>41.1<br>AST<br>40.5	188.3		A: 95.0<br>(57/60)<br>B: 5.0<br>(3/60)		1300	80.0<br>(48/60)		11.5<br>5.3-21.2
Arkadopoulos, 2012[17]	MH	NA	NA	NA	NA	NA	NA	NA	NA	NA	A: 100<br>(16/16)<br>A: 100<br>(20/20)	NA	NA	NA	NA	4<br>2.0-6.0<br>3.5<br>2.5-5.0
Chen, 2008[13]	MH	NA	84.0<br>(215/256)	23.0<br>(59 /256)	14.1<br>(36/256,<br>HBV 36)	NA	NA	NA	NA	NA	A: 93.0<br>(238/256)<br>B: 7.0<br>(18 /256)	NA	4200	84.4<br>(216<br>/256)	NA	7.2<br>NA
Chen, 2007[10]	MH	44.9<br>(40/89)	83.1<br>(74/89)	NA	84.3<br>(75/89,<br>HBV 75)	12.1	38.0	NA	NA	NA	A: 87.6<br>(78/89)<br>B: 12.4<br>(11/89)	NA	2838	86.5<br>(77 /89)	60.7<br>(54/89)	10.3<br>NA
		42.7<br>(67/157)	84.1<br>(132/157)		83.4<br>(131/157,<br>HBV131)	11.8	39.0				A: 90.4<br>(142/157)<br>B: 9.6<br>(15/157)		2335	83.4<br>(131/157)	56.1<br>(88/157)	9.9<br>NA

Dai, 2008[15]	MH	NA	NA	NA	100 (17/17, most for HBV)	NA	NA	NA	NA	NA	A: 100 (17/17)	NA	NA	NA	NA	12.4 4.0-21.0
Miao, 2009[21]	MH	NA	NA	NA	100 (47/47, most for HBV)	NA	NA	NA	NA	NA	A: 100 (47/47)	NA	NA	NA	NA	11.2 4.0-23.0
Hasegawa, 1989[18]	MH	NA	NA	NA	NA	NA	31.7	AST 49.9	NA	NA	NA	19.9	NA	NA	NA	9.5 3.0-17.0
Gallagher, 2013[11]	MH	61.9 (13/21)	100 (21/21)	NA	85.7 (18/21, HBV 16 HCV 2)	13.0	43.0	AST 47.0	194.0	NA	A: 100 (21/21)	9.9	NA	90.5 (19/21)	NA	7.0 2.3-11.0
Wu, 1999[8]	MH	66.7 (10/15)	73.3 (11/15)	20.0 (3/15)	100 (15/15, HBV 13 HCV 2)	NA	NA	NA	NA	NA	A: 93.3 (14/15) B: 6.7 (1/15)	NA	NA	73.3 (11/15)	26.7 (4/15)	12.8 8.5-18.0
	EH	NA	NA	NA	NA	NA	NA	NA	NA	NA	NA	NA	NA	NA	NA	NA
Scudamore, 2000[9]	MH	NA	16.7 (3/18)	NA	27.8 (5/18, HBV 5)	NA	NA	NA	NA	NA	NA	NA	NA	NA	NA	NA
	EH	NA	NA	NA	NA	NA	NA	NA	NA	NA	NA	NA	NA	NA	NA	NA

Qiu, 2013[1]	MH	NA	72.3 (211/292)	NA	73.3 (217/292, HBV 214 HCV 3)	14.2	42.9	ALT 51.1	146.5	11.5	A: 94.5 (276/292) B: 5.5 (16/292)	6.4	NA	82.2 (240/292)	NA	6.9<br>NA
	EH	NA	61.6 (85/138)	NA	68·8 (95/138, HBV 95)	15.5	40.8	ALT 45·7	155.5	11.4	A: 90.6 (125/138) B: 9.4 (13/138)	6.2	NA	76.1 (105/138)	NA	7.2
Chen, 2014[6]	MH	25.4 (30/118)	89.8 (106/118)	8.5 (10/118)	97.5 (115/118, HBV 100 HCV 15)	NA	NA	NA	NA	NA	A: 100 (118/118)	5.3	NA	NA	NA	8.6<br>2.5-13.6
	EH	29.8 (14/47)	85.1 (40/47)	17.0 (8/47)	97.9 (46/47, HBV 41 HCV 5)	NA	NA	NA	NA	NA	A: 100 (47/47)	5.2	NA	NA	NA	8.4
		30.3 (10/33)	84.8 (28/33)	12.1 (4/33)	100 (33/33, HBV 28 HCV 5)						A: 100 (33/33)	4.9				8.2
Yang, 2014[7]	MH	53.2 (84/158)	81.0 (128/158)	NA	90.5 (143/158, HBV143)	23.3	41.1	ALT 58.3	165.5	11.5	A: 83.5 (132/158) B: 16.5 (26/158)	NA	NA	48.7 (77 /158)	53.8 (85/158)	7.5<br>NA

		57.8<br>(111/192)	79.7<br>(153/192)		89.6<br>(172/192,<br>HBV172)	21.6	42.1	ALT<br>51.9	171.8	11.3	A: 86.5<br>(166/192)<br>B: 13.5<br>(26/192)			43.8<br>(84/192)	55.7<br>(107/192)	3.2<br>NA
	EH	56.1<br>(194/346)	78.6<br>(272/346)	NA	87.6<br>(303/346,<br>HBV303)	21.5	40.1	ALT<br>53.1	176.4	11.6	A: 82.1<br>(284/346)<br>B: 17.9<br>(62/346)	NA	NA	40.8<br>(141/346)	47.1<br>(163/346)	8.1
Hu, 2003[4]	MH	23.1<br>(12/52)	46.2<br>(24/52)	NA	46.2<br>(24/52,<br>HCV 24)	NA	NA	NA	NA	NA	NA	17.4	NA	90.4<br>(47/52)	75.0<br>(39/52)	NA
	EH	42.6<br>(26/61)	38.1<br>(24/63)	NA	20.6<br>(13/63,<br>HCV 13)	NA	NA	NA	NA	NA	NA	12.4	NA	79.4<br>(50/63)	52.4<br>(33/63)	NA
Cheng, 2012[5]	MH	47.6<br>(30/63)	47.6<br>(30/63)	11.1<br>(7/63)	92.1<br>(58/63,<br>HBV 43<br>HCV 15)	13.7	40.5	ALT<br>41.0<br>AST<br>38.00	198.0	11.8	A: 88.9<br>(56/63)<br>B: 11.1<br>(7/63)	8.6	58	NA	71.4<br>(45/63)	7.2<br>NA
	EH	46.3<br>(19/41)	36.6<br>(15/41)	7.3<br>(3/41)	85.4<br>(35/41,<br>HBV 27<br>HCV 8)	13.7	42.0	ALT<br>49.0<br>AST<br>49.0	179.5	11.9	A: 90.2<br>(37/41)<br>B: 7.3<br>(3/41)	8.1	82	NA	63.4<br>(26/41)	8.5

Polled value	MH	44.1	79.5	17.7	85.5 HBV73.1 HCV11.7	15.9	39.7	ALT 47.0 AST 38.5	173.9	11.6	A:92.4 B:7.6	11.4	1991.9	80.6	58.2	8.6
	95% CI	33.4-55.3	70.3-86.4	9.0-31.9	71.0-93.5 HBV 55.0-85.8 HCV 5.0-24.9	12.4-19.4	37.7-41.6	ALT 38.0-56.0 AST 29.7-47.3	158.2-189.7	11.2-11.9	A:89.2-94.7 B:5.3-10.8	5.7-17.0	654.6-3329.2	66.6-89.6	49.1-66.7	7.3-10.0
	EH	44.0	62.4	12.2	78.7 HBV78.9 HCV17.2	17.0	40.8	ALT 50.1 AST -	170.8	11.7	A:89.5 B:10.5	7.9	-	66.6	52.0	8.2
	95% CI	31.6-57.2	42.9-78.5	6.3-22.2	51.2-92.9 HBV 64.7-88.4 HCV 12.4-23.5	11.8-22.1	39.5-42.0	ALT 45.4-54.9 AST -	156.9-184.6	11.4-11.9	A:80.2-94.7 B:5.3-19.8	4.0-11.9	-	36.9-87.2	43.3-60.6	7.8-8.5

MH=mesohepatectomy; EH=hemi- or extended- hemihepatectomies; 95% CI=95% confidence interval; NA=not available; TACE=transarterial chemoembolization; TACI=transarterial chemoinfusion; ALT=alanine aminotransferase; AST=aspartate aminotransferase; PLT=platelet; PT=prothrombin time; ICG-R15=indocyanine green retention rate at 15 min; AFP=alpha-fetoprotein; HBV=hepatitis B virus; HCV= hepatitis C virus.

Supplementary table 2. Extracted data and pooled values of intraoperative variables for the 20 included studies

Ref. Year	Type of Operation	Segments of Resection (number of patients)	Operation time(Mean/ Median,min) (Range, min)	Occlusion Time(Mean/ Median,min)(Range, min)	Transection Time(Mean, min)	Blood Loss (Mean/Median,ml)(Range, ml)	Rate of Transfusion Required (%)	Volume of Transfusion (Mean/Median,ml)(Range, ml)	Weight of Resected Liver(Mean/ Median, g) (Range, g)	Section Margin (Mean/Median, cm) or (≥1cm%)or(R0%)
Zuo, 2014[3]	MH	IV, V and VIII: 9<br>IVb,V and VIII: 8<br>IVa, V and VIII: 4<br>I, IV, V and VIII: 3	238<br>NA	23.0<br>15.0-60.0	NA	480<br>200-2200	NA	NA	NA	NA
Mehrabi, 2008[14]	MH	IV, V and VIII: 48	238<br>65-480	NA	NA	1120<br>100-5000	66.7<br>(32/48)	738<br>200-2400	NA	NA
Chouillard, 2003[20]	MH	IV, V and VIII: 16	280<br>180-420	NA	NA	NA	NA	NA	NA	NA
Lee, 2008[19]	MH	IV, V and VIII: 27	330<br>215-669	NA	NA	1400<br>550-7000	NA	345<br>0-3200	300<br>147-483	1.5*
Giuliante, 2008[16]	MH	IV, V and VIII: 18	448<br>240-792	60.0<br>16.0-125.0	NA	NA	27.8<br>(5/18)	400<br>200-800	NA	0.6*
Chen, 2006[12]	MH	V and VIII: 8<br>IV, V and VIII: 49<br>V and VIII: 7<br>IV, V and VIII: 53	124.5<br>88–243<br>133<br>95-271	13.5<br>7.0-28.0<br>12.6<br>6.0-30.0	NA	770<br>230-3800<br>420<br>130-980	46.6<br>(27/58)<br>13.3<br>(8/60)	NA	761<br>430-2150<br>816<br>550-2360	NA

Arkadopoulos, 2012[17]	MH	I, IV, V and VIII: 36	180<br>120-230<br>150<br>115-190	46.0<br>36.0-61.0<br>28.0<br>22.0-45.0	NA	650<br>300-1100<br>400<br>200-700	NA	440<br>0-800<br>240<br>0-1000	NA	NA
Chen, 2008[13]	MH	IV, V and VIII: 248<br>I, IV, V and VIII: 8	174<br>NA	10.8<br>NA	NA	510<br>NA	20.3<br>(52/256)	NA	230<br>NA	57.8†
Chen, 2007[10]	MH	IV, V and VIII: 82<br>I, IV, V and VIII: 7	177<br>NA	15.2<br>NA	21.0	790<br>NA	44.9<br>(40/89)	NA	NA	19.1†
		IV, V and VIII: 141<br>I, IV, V and VIII: 16	115<br>NA	11.3<br>NA	16.0	420<br>NA	24.8<br>(39/157)			16.6†
Dai, 2008[15]	MH	IV, V and VIII: 17	245<br>190-330	NA	NA	NA	17.6<br>(3/17)	NA<br>300-1500	NA	41.2†
Miao, 2009[21]	MH	IV, V and VIII: 47	211<br>125-330	50.0<br>11.0-107.0	NA	580<br>180-4500	29.8<br>(14/47)	NA<br>300-4000	NA	27.7†
Hasegawa, 1989[18]	MH	IV, V and VIII: 13<br>IV, V, VIII ,VII+part of II and III: 1<br>V, VIII+part of IV: 2	NA<br>300-660	NA	NA	2850<br>600-7500	NA	NA	NA<br>370-1440	NA
Gallagher, 2013[11]	MH	IV, V and VIII: 21	627<br>240-1149	NA	NA	1590<br>120-3750	57.1<br>(12/21)	NA	NA	0.5*<br>100§
Wu, 1999[8]	MH	IV, V and VIII: 15	474<br>342-660	131.0<br>68.4-184.4	NA	2450<br>720-5350	66.7<br>(10/15)	1100<br>0-3000	859<br>290-1750	0.5*
	EH	extended right lobectomy: 14<br>extended left lobectomy: 11	348<br>270-630	58.4<br>36-108.5	NA	1863<br>520-4360	72.0<br>(18/25)	768<br>0-3000	NA	NA
Scudamore, 2000[9]	MH	IV, V, VIII±I: 18	238<br>115-435	45.0<br>19.0-74.0	NA	914<br>NA	44.4<br>(8/18)	NA	560<br>NA	NA

	EH	extended right lobectomy: 32<br>extended left lobectomy: 11	304<br>NA	39.0<br>NA	NA	1628<br>NA	NA	NA	1500<br>NA	NA
Qiu, 2013[1]	MH	V, IVb+part of IVa and VIII: 88<br>IVa, VIII+part of V and IVb: 56<br>Irregular resection: 59<br>IV, V and VIII± I: 19<br>IV, V and VIII ± I: 70	259<br>NA	NA	NA	634<br>NA	33.9<br>(99 /292)	820<br>NA	NA	NA
	EH	extended hemihepatectomy: 138	211<br>NA	NA	NA	570<br>NA	24.6<br>(34/138)	750<br>NA	NA	NA
Chen, 2014[6]	MH	IV, V and VIII±I: 118	244<br>100-450	30.3<br>0.0-90.0	NA	NA	34.7<br>(41/118)	456<br>0-2400	NA	66.9†
	EH	extended right lobectomy: 47	225<br>165-370	28.7<br>5-60	NA	NA	36.2<br>(17/47)	532<br>0-3200	NA	83.0†
		extended left lobectomy: 33	219<br>162-290	25.4<br>0-35			30.3<br>(10/33)	493<br>0-2600		83.0†
Yang, 2014[7]	MH	IV, V, and VIII: 350	NA	17.9<br>NA	15.2	951<br>NA	37.3<br>(59/158)	NA	NA	52.5†
				16.3<br>NA	14.2	903<br>NA	31.8<br>(61/192)			64.1†
	EH	hemihepatectomy or less: 346	NA	15.8<br>NA	13.9	855<br>NA	26.3<br>(91/346)	NA	NA	61.6†
Hu, 2003[4]	MH	(IV, V and VIII) or (V and VIII): 52	265<br>NA	NA	NA	NA	NA	1030<br>NA	NA	57.7†
	EH	Hemihepatectomy or extended: 63	264<br>NA	NA	NA	NA	NA	440<br>NA	NA	74.6†

Cheng, 2012[5]	MH	I, IV, V and VIII: 63	230<br>NA	NA	NA	500<br>NA	12.7<br>(8/63)	600<br>NA	227<br>NA	7.4†
	EH	Hemihepatectomy or extended: 41	316<br>NA	NA	NA	750<br>NA	NA	600<br>NA	590<br>NA	21.1†
	MH	Resection of Segment I%: 5.1	245	29.5	16.3	720	34.0	726	526	40.1†
	95% CI	Resection of Segment I%:2.1-12.1	219-272	26.2-32.8	13.2-19.3	578-861	28.1-40.5	527-925	179-872	26.0-56.1†
Polled value	EH	Hemihepatectomy%:38.4<br>Extended:61.6<br>Left extended%:36.9<br>Right extended%:63.1	274	35.0	-	`1001	35.1	592	1029	62.3†
	95% CI	Hemihepatectomy%:1.1-97.2<br>Extended%:2.8-98.9<br>Left extended%:26.7-48.4<br>Right extended%:51.6-73.3	236-313	17.3-52.7	-	743-1260	23.7-48.5	474-709	138-1920	40.3-80.1†

MH=mesohepatectomy; EH=hemi- or extended- hemihepatectomies; 95% CI=95% confidence interval; NA=not available; R0=tumor-free margins; *=mean or median section margin; †=section margin ≥1cm%; §=R0%.

Supplementary table 3. Extracted data and pooled values of postoperative variables for the 20 included studies

Ref. Year	Type of Operation	Differentiation(Edmondson-Steiner grade) (%)	Overall Morbidity(%)	Recurrence(%)	Reoperation(%)	Rate of Liver Failure (%)	Mortality (%)	Causes for Mortalityand number of patients	Length of Postoperative Stay(Mean/Median,d)(Range, d)*	Median OS(m) and Median DFS(m)*	1-year OS and DFS (%)*	3-year OS and DFS (%)*	5-year OS and DFS (%)*	6-year OS and DFS (%)*
Zuo, 2014[3]	MH	NA	33.3<br>(8/24)	NA	NA	NA	NA	NA	NA	NA	76.0<br>NA	46.0<br>NA	NA	NA
Mehrabi, 2008[14]	MH	NA	27.1<br>(13/48)	NA	NA	2.1<br>(1/48)	2.1<br>(1/48)	liver failure:1	15.8<br>(6-104)	NA	NA	NA<br>47.9	NA	NA
Chouillard, 2003[20]	MH	NA	18.8<br>(3/16)	NA	NA	NA	0.0<br>(0/16)	-	NA	NA	NA	NA	NA	NA
Lee, 2008[19]	MH	NA	44.4<br>(12/27)	29.6<br>(8/27)	NA	NA	NA	NA	NA	NA	NA	NA	NA	NA
Giuliante, 2008[16]	MH	NA	33.3<br>(6/18)	22.2<br>(4/18)	NA	NA	0.0<br>(0/18)	-	18.0	NA	NA	NA	NA	NA
Chen, 2006[12]	MH	NA	29.3<br>(17/58)<br>31.7<br>(19/60)	NA	NA	3.4<br>(2/58)<br>3.3<br>(2/60)	1.7<br>(1/58)<br>0.0<br>(0/60)	liver failure:1<br>-	NA	NA	NA	NA	NA	NA

Arkadopoulos, 2012[17]	MH	NA	37.5<br>(6/16)<br>45<br>(9/20)	NA	NA	NA	0.0<br>(0/16)<br>0.0<br>(0/20)	-	NA	NA	NA	NA	NA	NA
Chen, 2008[13]	MH	NA	28.1<br>(72/256)	82.8<br>(212/256)	NA	NA	0.4%<br>(1/256)	NA	NA	36.9<br>16.0	77.0<br>59.1	49.8<br>28.8	35.1<br>17.0	NA
Chen, 2007[10]	MH	NA	34.8<br>(31/89)	79.8<br>(71/89)	0.0<br>(0/89)	2.2<br>(2/89)	3.4<br>(3/89)	liver failure:2<br>sepsis:1	NA	57.0<br>36.0	87.1<br>75.0	62.9<br>46.2	46.2<br>31.8	NA
			24.2<br>(38/157)	86.6<br>(136/157)	0.0<br>(0/157)	0.6<br>(1/157)	0.6<br>(1/157)	liver failure:1	NA	41.0<br>28.0	82.2<br>69.6	54.4<br>38.0	31.7<br>16.5	NA
Dai, 2008[15]	MH	NA	47.1<br>(8/17)	NA	NA	0.0<br>(0/17)	0.0<br>(0/17)	-	NA	NA	NA	NA	NA	NA
Miao, 2009[21]	MH	NA	34.0<br>(16/47)	NA	NA	0.0<br>(0/47)	0.0<br>(0/47)	-	NA	NA	NA	NA	41.0<br>NA	NA
Hasegawa, 1989[18]	MH	NA	37.5<br>(6/16)	68.8<br>(11/16)	6.3<br>(1/16)	6.3<br>(1/16)	6.3<br>(1/16)	liver failure:1	NA	34.0<br>NA	81.3<br>NA	43.8<br>NA	37.5<br>NA	25.0<br>NA
Gallagher, 2013[11]	MH	NA	42.9<br>(9/21)	33.3<br>(7/21)	23.8<br>(5/21)	4.8<br>(1/21)	4.8<br>(1/21)	multiorgan<br>failure:1	NA	32.3<br>15.5	90.5<br>65.0	66.8<br>34.8	66.8<br>34.8	NA
Wu, 1999[8]	MH	NA	20.0<br>(3/15)	93.3<br>(14/15)	NA	0.0<br>(0/15)	0.0<br>(0/15)	-	NA	33<br>15.0	67.0<br>53.0	44.0<br>31.0	30.0<br>20.0	30.0<br>21.0
	EH	NA	36.0<br>(9/25)	NA	NA	8.0<br>(2/25)	4.0<br>(1/25)	liver failure:1	NA	NA	NA<br>38.0	NA<br>19.0	NA	NA<br>9.0
Scudamore, 2000[9]	MH	NA	61.1<br>(11/18)	NA	NA	NA	0.0<br>(0/18)	-	9.0	NA	NA	NA	NA	NA

Qiu, 2013[1]	EH	NA	74.4<br>(32/43)	NA	NA	NA	0.0<br>(0/43)	-	16.0	NA	NA	NA	NA	NA
	MH	NA	19.9<br>(58/292)	NA	0.7<br>(2/292)	NA	0.7<br>(2/292)	uncontrollable<br>bleeding: 1<br>sepsis: 1	NA	NA	NA	NA	NA	NA
Chen, 2014[6]	EH	NA	52.2<br>(72/138)	NA	0.0<br>(0/138)	NA	1.4<br>(2/138)	liver failure:1	NA	NA	NA	NA	NA	NA
	MH	I: 24.6(29/118)<br>II: 56.8(67/118)<br>III: 18.6(22/118)	37.3<br>(44/118)	NA	NA	1.7<br>(2/118)	2.5<br>(3/118)	liverfailure: 2<br>severe<br>pulmonary<br>infection: 1	17.7<br>(7-66)	30.0<br>15.3	67.8<br>53.4	45.5<br>30.5	28.9<br>16.9	NA
Yang, 2014[7]	EH	I: 36.2(17/47)<br>II: 46.8(22/47)<br>III: 17.0(8/47)	55.3<br>(26/47)	NA	NA	10.6<br>(5/47)	8.5<br>(4/47)	liver failure: 3<br>postoperative<br>bleeding: 1	17.2<br>(11-53)	30.0<br>22.7	73.8<br>61.2	44.5<br>37.5	30.3<br>27.4	NA
		I: 33.3(11/33)<br>II: 45.5(15/33)<br>III: 21.2(7/33)	24.2<br>(8/33)			6.1<br>(2/33)	3.0<br>(1/33)	liver failure:1	15.6<br>(10-24)					
	MH	I+II: 54.4(86/158)<br>III+IV: 45.6(72/158)	13.9<br>(22/158)	69.6<br>(110/158)	NA	NA	0.0<br>(0/158)	-	NA	33.0<br>21.0	79.0<br>58.0	47.0<br>37.0	30.0<br>26.0	NA
		NA	11.5<br>(22/192)	61.5<br>(118/192)			0.0<br>(0/192)			37.0<br>21.0	80.0<br>68.0	52.0<br>37.0	31.0<br>25.0	
Hu, 2003[4]	EH	NA	11.3<br>(39/346)	64.7<br>(224/346)	NA	NA	0.6<br>(2/346)	liver failure:2	NA	40.5<br>24.0	84.0<br>65.0	55.0<br>37.0	32.0<br>23.0	NA
	MH	I+II: 65.4(34/52)<br>III+IV: 34.6(18/52)	17.3<br>(9/52)	61.5<br>(32/52)	5.8<br>(3/52)	NA	0.0<br>(0/52)	-	NA	51.0<br>23.0	89.0<br>63.0	75.0<br>29.0	70.0<br>23.0	NA

Cheng, 2012[5]	EH	I+II: 46.0(29/63)<br>III+IV: 54.0(34/63)	19.0<br>(12/63)	54.0<br>(34/63)	12.7<br>(8/63)	3.2<br>(2/63)	3.2<br>(2/63)	liver failure:2	NA	47.0<br>25.0	94.0<br>74.0	75.0<br>44.0	59.0<br>42.0	NA
	MH	I+II: 39.7(25/63)<br>III+IV: 60.3(38/63)	12.7<br>(8/63)	61.9<br>(39/63)	6.3<br>(4/63)	3.2<br>(2/63)	7.9<br>(5/63)	multiorgan<br>failure:5	10.0	42.0<br>9.8	87.5<br>50.0	57.0<br>27.0	53.1<br>15.0	NA
	EH	I+II: 34.1(14/41)<br>III+IV: 65.9(27/41)	14.6<br>(6/41)	41.5<br>(17/41)	NA	4.9<br>(2/41)	7.3<br>(3/41)	multiorgan<br>failure:3	11.2	35.0<br>11.2	89.5<br>50.0	51.0<br>38.9	66.2<br>38.9	NA
Polled value	MH	I+II:61.4<br>III+IV:38.6	29.2	62.6	3.9	2.5	2.0	52.2‡	13.5	38.2<br>16.4	80.8<br>60.1	54.0<br>33.2	42.5<br>19.7	27.3<br>-
	95% CI	I+II:42.9-77.1<br>III+IV:22.9-57.1	24.1-34.8	49.6-74.0	1.1-12.7	1.5-4.0	1.2-3.3	28.6-75.0‡	8.7-18.2	34.0-42.8<br>12.6-21.3	76.1-85.4<br>55.0-65.3	47.8-60.2<br>29.1-37.4	33.9-51.1<br>16.2-23.1	11.6-42.9<br>-
	EH	I+II:55.5<br>III+IV:44.5	32.9	55.0	2.9	6.7	2.8	71.8‡	14.2	37.7<br>19.8	85.8<br>60.1	56.6<br>36.3	46.0<br>31.3	-
	95% CI	I+II:26.8-80.9<br>III+IV:19.1-73.2	17.0-54.0	41.4-67.9	0.1-51.6	3.9-11.2	1.3-5.9	44.5-89.0‡	9.7-18.7	30.4-46.7<br>16.7-23.4	78.3-93.2<br>51.0-69.2	44.9-68.3<br>30.3-42.4	29.7-62.2<br>22.0-40.6	-

MH=mesohepatectomy; EH=hemi- or extended- hemihepatectomies; 95% CI=95% confidence interval; NA=not available; OS=overall survival; DFS=disease free survival;

*=the value of upper line and lower line for OS and DFS respectively; ‡=liver failure%

Supplementary table 4. Indications Reported for MH and EH

Type of Indication	MH<br>Number reported (*n*=1782)	MH<br>Frequency (%)	EH<br>Number reported (*n*=736)	EH<br>Frequency (%)	*p*
**Primary malignant diseases**	1701	95.5	704	95.7	0.828
Hepatocellular carcinoma	1683	94.4	687	93.3	0.285
Cholangiocarcinoma	9	0.5	10	1.4	0.024
Gallbladder carcinoma	7	0.4	7	1	0.087
Hepatosarcoma	2	0.1	-	-	-
**Metastases**	66	3.7	28	3.8	0.904
Colorectal	40	2.2	22	3	0.273
Pancreas	3	0.2	-	-	-
Kidney	2	0.1	-	-	-
Others	9	0.5	-	-	-
Not Specified	12	0.7	6	0.8	0.701
**Benign diseases**	15	0.8	4	0.5	0.252
Focal nodular hyperplasia	10	0.6	3	0.4	0.625
Hemangioma	2	0.1	-	-	-
Gastrointestinal stromal tumor	1	0.1	-	-	-
Others	2	0.1	-	-	-
Not Specified	-	-	1	0.1	-

MH=mesohepatectomy; EH=hemi- or extended- hemihepatectomies

Supplementary table 5. Postoperative Complications Reported for MH and EH

Type of complication	MH		EH		*p*
	Number reported (*n*=481)	Frequency (%)	Number reported (*n*=178)	Frequency (%)	
**Surgical complications**	113	23.5	47	26.4	0.439
Bilioma/bile leakage	65	13.5	12	6.7	0.016
Wound infection	33	6.9	28	15.7	0
Abdomen abscess	14	2.9	6	3.4	0.76
Others	1	0.2	1	0.6	0.47
**Medical complications**	368	76.5	131	73.6	0.439
Pleural effusion	166	34.5	29	16.3	0
Ascites	87	18.1	32	18.0	0.974
Pulmonary infection	33	6.9	14	7.9	0.656
Delayed jaundice	14	2.9	28	15.7	0
Liver failure	14	2.9	13	7.3	0.012
Haemorrhage/Stress ulcer bleeding	13	2.7	6	3.4	0.646
Others	41	8.5	9	5.1	0.184

Supplementary table 6. Postoperative Tumor Recurrence Reported for MH and EH

Type of Recurrence	MH		EH		*P*
	Number reported (*n*=742)	Frequency (%)	Number reported (*n*=250)	Frequency (%)	
**Intrahepatic recurrence**	585	78.8	194	77.6	0.679
Single lesion	70	9.4	4	1.6	-
Multiple lesion	153	20.6	9	3.6	-
Not Specified	362	48.8	181	72.4	-
**Extrahepatic recurrence**	157	21.2	56	22.4	0.679
Lung metastasis	47	6.3	-	-	-
Other organs metastasis	42	5.7	-	-	-
Not Specified	68	9.2	56	22.4	-

MH=mesohepatectomy; EH=hemi- or extended- hemihepatectomies

Supplementary table 7. Methods of hepatic blood occlusion Reported for MH

**Type of Occlusion Method**	**Feature**	**Intermittent%**	**Number reported (*n*=1585)**	**Frequency (%)**
Occlusion of hepatic blood inflow /Pringle maneuver [1, 3, 5, 7-9, 12, 14-16, 18, 23]	Clamping of the hepatic pedicle	97.9	985	62.1
Hemihepatic vascular occlusion /selective inflow blood occlusion [5-9, 13, 17, 20-23]	Clamping of the ipsilateral hepatic artery and portal vein	96.3	354	22.3
Hepatic vascular exclusion (HVE) [3]	Sequential clamping of portal triad and the inferior vena cava(IVC) both above and below the liver	100	3	0.2
Modified technique of hepatic vascular exclusion (MTHVE) [14, 15]	Sequential clamping of portal triad and the IVC below the liver	100	201	12.7
Extraparenchymal control of hepatic veins/Selective hepatic vascular exclusion (SHVE) [18, 19]	Sequential clamping of hepatic pedicle, right hepatic vein (RHV), the left hepatic vein (LHV) and the middle hepaticvein (MHV)	100	22	1.4
Sequential hemihepatic vascular control (SHHVC) [19]	Sequential clamping of left portal pedicle, left and middle hepatic veins→left transection→Outflow and inflow restored→the same process on the right	-	20	1.3

Supplementary table 8. Terminology of MH

Year	First author	Terms	title of article
1961	Pack	middle hepatic lobectomy	Middle hepatic lobectomy for cancer [28]
1972	McBride	central hepatectomy	Cancer of the right lobe of the liver: a variety of operative procedures [34]
1977	Foster	middle lobectomy	Solid liver tumors [29]
1987	Lin TY	middle hepatectomy	Role of surgery in the treatment of primary carcinoma of the liver: A 31-year experience [30]
1989	Hasegawa	central bisegmentectomy	Central bisegmentectomy of the liver: experience in 16 patients [20]
1998	Billingsley	Central hepatectomy/mesohepatectomy	Segment-oriented hepatic resection in the management of malignant neoplasms of the liver[31]
1999	Wu C C	Mesohepatectomy	Mesohepatectomy for centrally located hepatocellular carcinoma: an appraisal of a rare procedure [8]
2002	La Quaglia MP	Central hepatic resection	Central hepatic resection for pediatric tumors [32]
2008	Lee JG	central bisectionectomy	Central bisectionectomy for centrally located hepatocellular carcinoma [21]

Supplementary forest plots [posted as supplied by author]

Study or Subgroup	MH Mean	MH SD	MH Total	EH Mean	EH SD	EH Total	Weight	Mean Difference IV, Random, 95% CI
**1.2.1 age < 60 years**								
Qiu, 2013	634	585	292	570	507	138	31.3%	64.00 [-43.97, 171.97]
Wu, 1999	2,450	1,381	15	1,863	1,487	25	5.5%	587.00 [-323.05, 1497.05]
Yang, 2014	925	358	350	855	484	346	32.7%	70.00 [6.70, 133.30]
**Subtotal (95% CI)**			**657**			**509**	**69.4%**	**70.33 [15.81, 124.84]**
Heterogeneity: Tau² = 0.00; Chi² = 1.25, df = 2 (P = 0.53); I² = 0%								
Test for overall effect: Z = 2.53 (P = 0.01)								
**1.2.2 age > 60 years**								
Cheng, 2012	63	500	481	750	1,037	41	20.5%	-687.00 [-1007.55, -366.45]
Scudamore, 2000	914	1,000	18	1,628	1,340	43	10.1%	-714.00 [-1325.41, -102.59]
**Subtotal (95% CI)**			**499**			**84**	**30.6%**	**-692.82 [-976.72, -408.92]**
Heterogeneity: Tau² = 0.00; Chi² = 0.01, df = 1 (P = 0.94); I² = 0%								
Test for overall effect: Z = 4.78 (P < 0.00001)								
**Total (95% CI)**			**1156**			**593**	**100.0%**	**-137.78 [-370.21, 94.65]**
Heterogeneity: Tau² = 41953.54; Chi² = 28.03, df = 4 (P < 0.0001); I² = 86%								
Test for overall effect: Z = 1.16 (P = 0.25)								
Test for subgroup differences: Chi² = 26.77, df = 1 (P < 0.00001), I² = 96.3%								

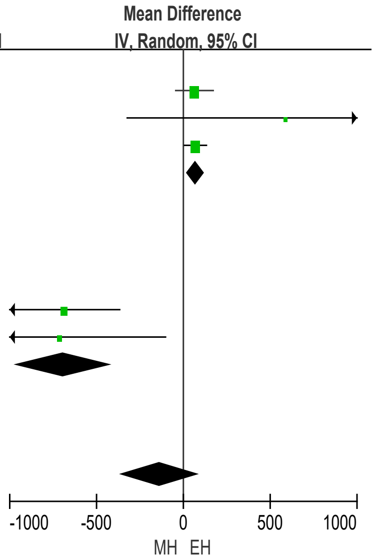

Fig A Subgroup analysis of blood loss by mean age of patients

Study or Subgroup	MH Events	MH Total	EH Events	EH Total	Weight	Odds Ratio M-H, Random, 95% CI
**2.2.1 age < 60 years**						
Chen, 2014	44	118	34	80	17.2%	0.80 [0.45, 1.44]
Qiu, 2013	58	292	72	138	18.4%	0.23 [0.15, 0.35]
Wu, 1999	3	15	9	25	9.1%	0.44 [0.10, 2.00]
Yang, 2014	44	350	39	346	18.2%	1.13 [0.72, 1.79]
**Subtotal (95% CI)**		**775**		**589**	**62.9%**	**0.56 [0.23, 1.38]**
Total events	149		154			
Heterogeneity: Tau² = 0.69; Chi² = 26.69, df = 3 (P < 0.00001); I² = 89%						
Test for overall effect: Z = 1.26 (P = 0.21)						
**2.2.2 age > 60 years**						
Cheng, 2012	8	63	6	41	11.9%	0.85 [0.27, 2.65]
Hu, 2003	9	52	12	63	13.6%	0.89 [0.34, 2.31]
Scudamore, 2000	11	18	32	43	11.6%	0.54 [0.17, 1.74]
**Subtotal (95% CI)**		**133**		**147**	**37.1%**	**0.76 [0.41, 1.42]**
Total events	28		50			
Heterogeneity: Tau² = 0.00; Chi² = 0.47, df = 2 (P = 0.79); I² = 0%						
Test for overall effect: Z = 0.86 (P = 0.39)						
**Total (95% CI)**		**908**		**736**	**100.0%**	**0.63 [0.34, 1.15]**
Total events	177		204			
Heterogeneity: Tau² = 0.47; Chi² = 28.08, df = 6 (P < 0.0001); I² = 79%						
Test for overall effect: Z = 1.51 (P = 0.13)						
Test for subgroup differences: Chi² = 0.30, df = 1 (P = 0.58), I² = 0%						

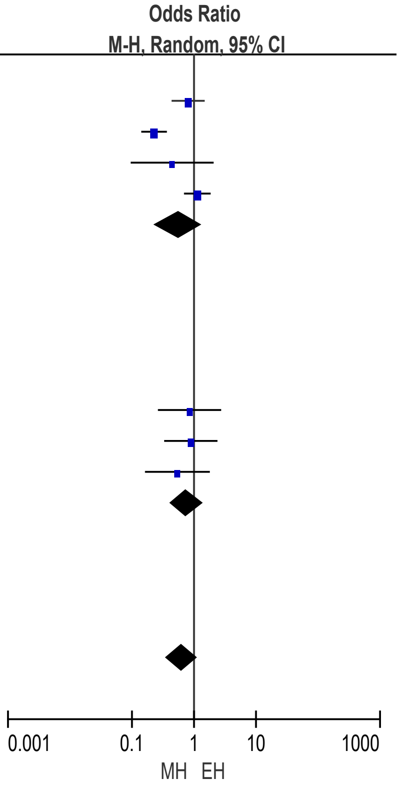

Fig B1 Subgroup analysis of morbidity rate by mean age of patients (the study Hu,2003 grouped to age>60years)(because study Hu,2003 failed to provide the age data)

Study or Subgroup	MH Events	MH Total	EH Events	EH Total	Weight	Odds Ratio M-H, Random, 95% CI
**2.3.1 age < 60 years**						
Chen, 2014	44	118	34	80	17.2%	0.80 [0.45, 1.44]
Hu, 2003	9	52	12	63	13.6%	0.89 [0.34, 2.31]
Qiu, 2013	58	292	72	138	18.4%	0.23 [0.15, 0.35]
Wu, 1999	3	15	9	25	9.1%	0.44 [0.10, 2.00]
Yang, 2014	44	350	39	346	18.2%	1.13 [0.72, 1.79]
**Subtotal (95% CI)**		**827**		**652**	**76.5%**	**0.61 [0.28, 1.31]**
Total events	158		166			
Heterogeneity: Tau² = 0.60; Chi² = 27.61, df = 4 (P < 0.0001); I² = 86%						
Test for overall effect: Z = 1.26 (P = 0.21)						
**2.3.2 age > 60 years**						
Cheng, 2012	8	63	6	41	11.9%	0.85 [0.27, 2.65]
Scudamore, 2000	11	18	32	43	11.6%	0.54 [0.17, 1.74]
**Subtotal (95% CI)**		**81**		**84**	**23.5%**	**0.68 [0.30, 1.54]**
Total events	19		38			
Heterogeneity: Tau² = 0.00; Chi² = 0.29, df = 1 (P = 0.59); I² = 0%						
Test for overall effect: Z = 0.92 (P = 0.36)						
**Total (95% CI)**		**908**		**736**	**100.0%**	**0.63 [0.34, 1.15]**
Total events	177		204			
Heterogeneity: Tau² = 0.47; Chi² = 28.08, df = 6 (P < 0.0001); I² = 79%						
Test for overall effect: Z = 1.51 (P = 0.13)						
Test for subgroup differences: Chi² = 0.04, df = 1 (P = 0.85), I² = 0%						

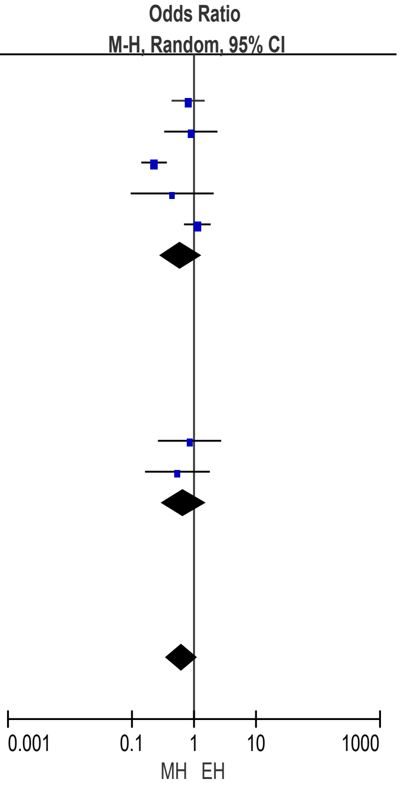

Fig B2 Subgroup analysis of morbidity rate by mean age of patients (the study Hu,2003 grouped to age<60years)

Study or Subgroup	MH Events	MH Total	EH Events	EH Total	Weight	Odds Ratio M-H, Fixed, 95% CI
**3.2.1 age < 60 years**						
Chen, 2014	3	118	5	80	32.8%	0.39 [0.09, 1.69]
Qiu, 2013	2	292	2	138	15.2%	0.47 [0.07, 3.36]
Wu, 1999	0	15	1	25	6.3%	0.53 [0.02, 13.77]
Yang, 2014	0	350	2	346	14.2%	0.20 [0.01, 4.11]
**Subtotal (95% CI)**		**775**		**589**	**68.4%**	**0.38 [0.14, 1.07]**
Total events	5		10			
Heterogeneity: Chi² = 0.26, df = 3 (P = 0.97); I² = 0%						
Test for overall effect: Z = 1.84 (P = 0.07)						
**3.2.2 age > 60 years**						
Cheng, 2012	5	63	3	41	18.9%	1.09 [0.25, 4.84]
Hu, 2003	0	52	2	63	12.7%	0.23 [0.01, 4.99]
Scudamore, 2000	0	18	0	43		Not estimable
**Subtotal (95% CI)**		**133**		**147**	**31.6%**	**0.75 [0.21, 2.63]**
Total events	5		5			
Heterogeneity: Chi² = 0.80, df = 1 (P = 0.37); I² = 0%						
Test for overall effect: Z = 0.45 (P = 0.65)						
**Total (95% CI)**		**908**		**736**	**100.0%**	**0.50 [0.23, 1.09]**
Total events	10		15			
Heterogeneity: Chi² = 1.77, df = 5 (P = 0.88); I² = 0%						
Test for overall effect: Z = 1.74 (P = 0.08)						
Test for subgroup differences: Chi² = 0.66, df = 1 (P = 0.42), I² = 0%						

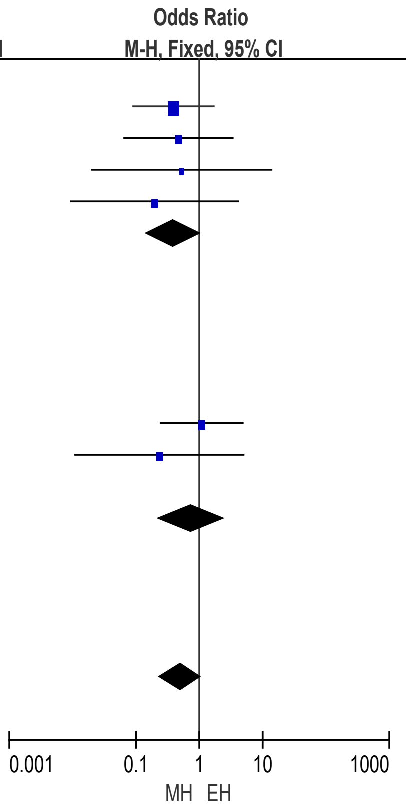

Fig C1 Subgroup analysis of mortality rate by mean age of patients (the study Hu,2003 grouped to age>60years)

Study or Subgroup	MH Events	MH Total	EH Events	EH Total	Weight	Odds Ratio M-H, Fixed, 95% CI
**3.3.1 age < 60 years**						
Chen, 2014	3	118	5	80	32.8%	0.39 [0.09, 1.69]
Hu, 2003	0	52	2	63	12.7%	0.23 [0.01, 4.99]
Qiu, 2013	2	292	2	138	15.2%	0.47 [0.07, 3.36]
Wu, 1999	0	15	1	25	6.3%	0.53 [0.02, 13.77]
Yang, 2014	0	350	2	346	14.2%	0.20 [0.01, 4.11]
**Subtotal (95% CI)**		**827**		**652**	**81.1%**	**0.36 [0.14, 0.95]**
Total events	5		12			
Heterogeneity: Chi² = 0.36, df = 4 (P = 0.99); I² = 0%						
Test for overall effect: Z = 2.07 (P = 0.04)						
**3.3.2 age > 60 years**						
Cheng, 2012	5	63	3	41	18.9%	1.09 [0.25, 4.84]
Scudamore, 2000	0	18	0	43		Not estimable
**Subtotal (95% CI)**		**81**		**84**	**18.9%**	**1.09 [0.25, 4.84]**
Total events	5		3			
Heterogeneity: Not applicable						
Test for overall effect: Z = 0.12 (P = 0.91)						
**Total (95% CI)**		**908**		**736**	**100.0%**	**0.50 [0.23, 1.09]**
Total events	10		15			
Heterogeneity: Chi² = 1.77, df = 5 (P = 0.88); I² = 0%						
Test for overall effect: Z = 1.74 (P = 0.08)						
Test for subgroup differences: Chi² = 1.51, df = 1 (P = 0.22), I² = 33.9%						

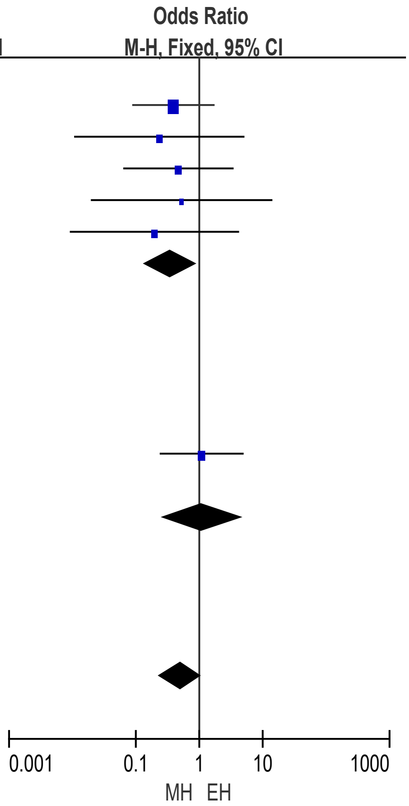

FigC2 Subgroup analysis of mortality rate by mean age of patients (the study Hu,2003 grouped to age<60years)

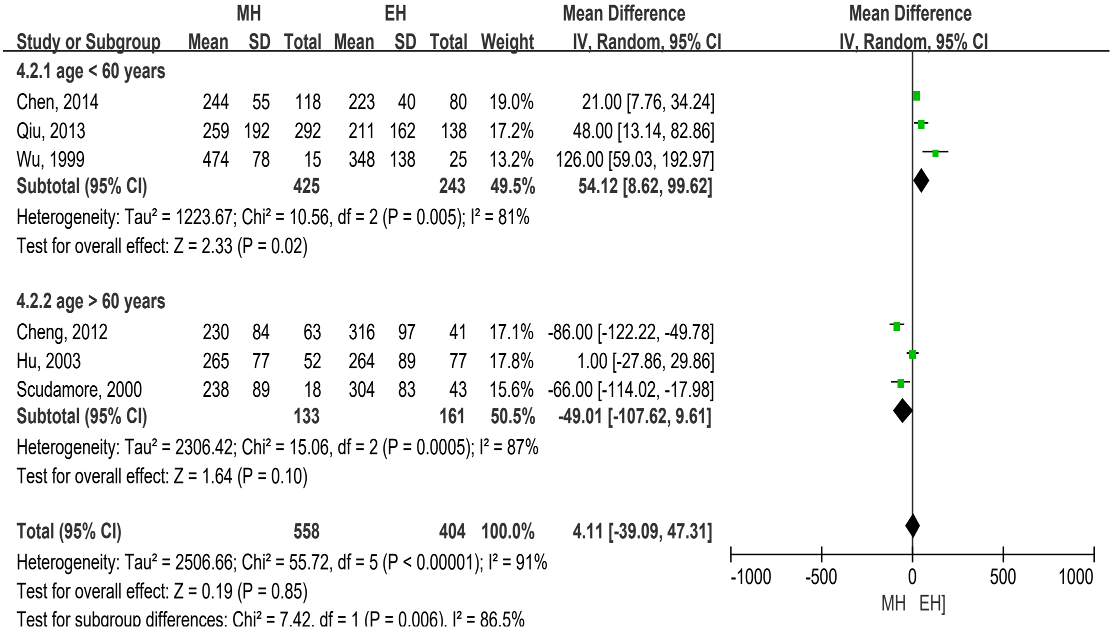

Fig D1 Subgroup analysis of operation time by mean age of patients (the study Hu,2003 grouped to age>60years)

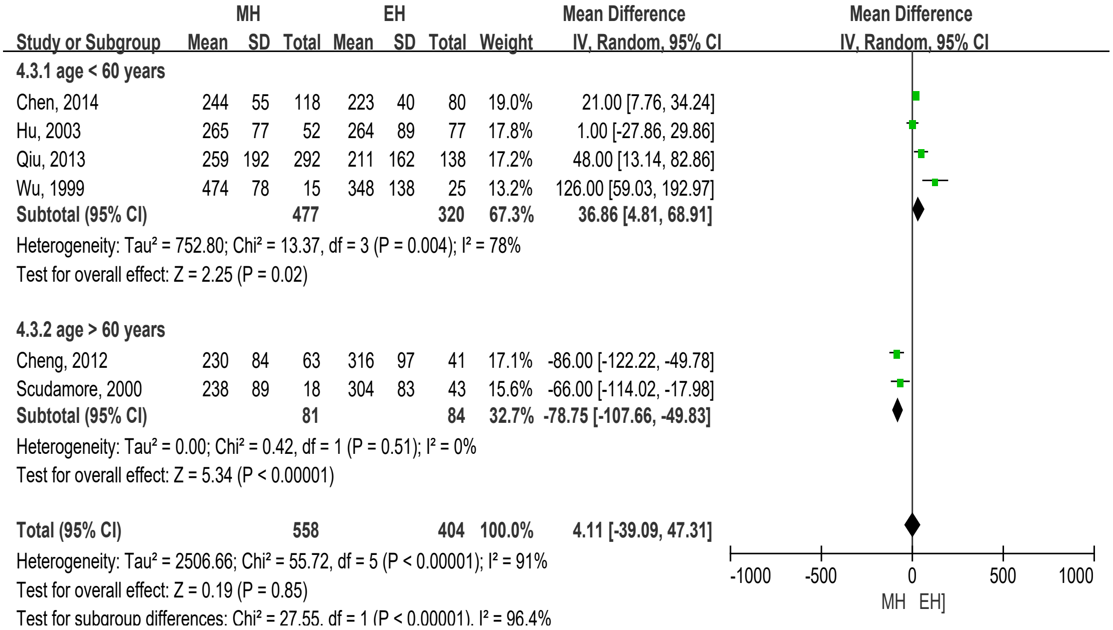

FigD2 Subgroup analysis of operation time by mean age of patients (the study Hu,2003 grouped to age<60years)

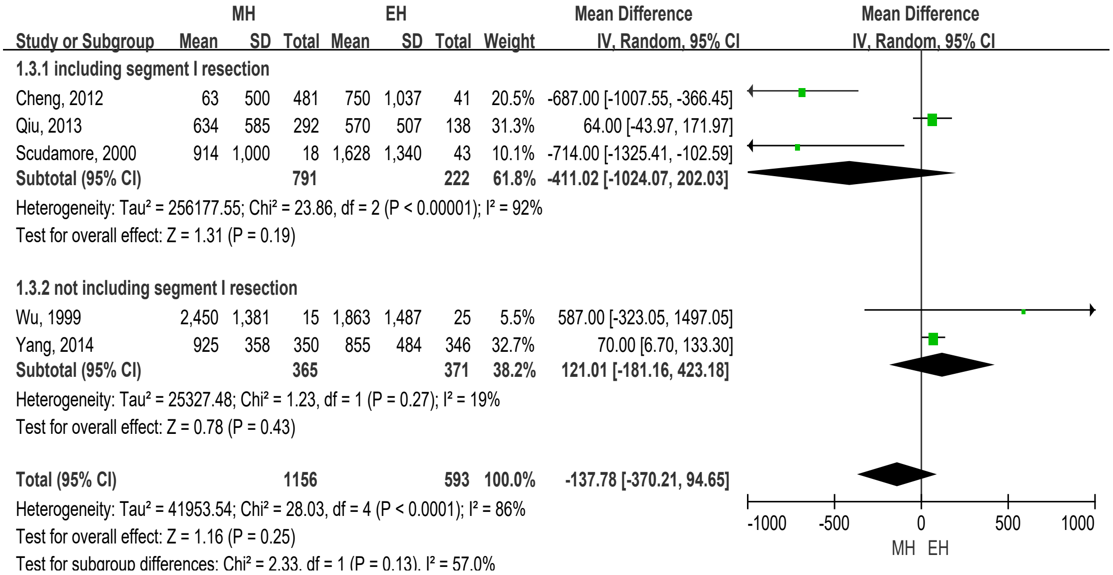

Fig E Subgroup analysis of blood loss by type of MH

Study or Subgroup	MH Events	MH Total	EH Events	EH Total	Weight	Odds Ratio M-H, Random, 95% CI
**2.4.1 including segment I resction**						
Chen, 2014	44	118	34	80	17.2%	0.80 [0.45, 1.44]
Cheng, 2012	8	63	6	41	11.9%	0.85 [0.27, 2.65]
Qiu, 2013	58	292	72	138	18.4%	0.23 [0.15, 0.35]
Scudamore, 2000	11	18	32	43	11.6%	0.54 [0.17, 1.74]
**Subtotal (95% CI)**		**491**		**302**	**59.1%**	**0.51 [0.23, 1.13]**
Total events	121		144			
Heterogeneity: Tau² = 0.48; Chi² = 13.85, df = 3 (P = 0.003); I² = 78%						
Test for overall effect: Z = 1.66 (P = 0.10)						
**2.4.2 not including segment I resection**						
Hu, 2003	9	52	12	63	13.6%	0.89 [0.34, 2.31]
Wu, 1999	3	15	9	25	9.1%	0.44 [0.10, 2.00]
Yang, 2014	44	350	39	346	18.2%	1.13 [0.72, 1.79]
**Subtotal (95% CI)**		**417**		**434**	**40.9%**	**1.02 [0.68, 1.51]**
Total events	56		60			
Heterogeneity: Tau² = 0.00; Chi² = 1.45, df = 2 (P = 0.49); I² = 0%						
Test for overall effect: Z = 0.08 (P = 0.94)						
**Total (95% CI)**		**908**		**736**	**100.0%**	**0.63 [0.34, 1.15]**
Total events	177		204			
Heterogeneity: Tau² = 0.47; Chi² = 28.08, df = 6 (P < 0.0001); I² = 79%						
Test for overall effect: Z = 1.51 (P = 0.13)						
Test for subgroup differences: Chi² = 2.32, df = 1 (P = 0.13), I² = 57.0%						

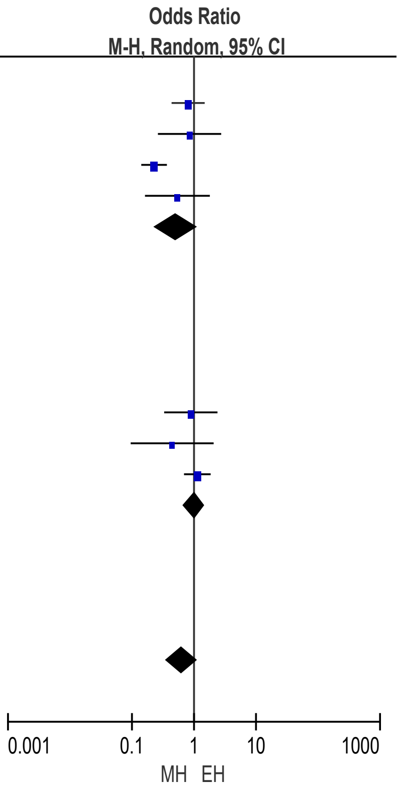

Fig F Subgroup analysis of morbidity rate by type of MH

Study or Subgroup	MH Events	MH Total	EH Events	EH Total	Weight	Odds Ratio M-H, Fixed, 95% CI
**3.4.1 including segment I resection**						
Chen, 2014	3	118	5	80	32.8%	0.39 [0.09, 1.69]
Cheng, 2012	5	63	3	41	18.9%	1.09 [0.25, 4.84]
Qiu, 2013	2	292	2	138	15.2%	0.47 [0.07, 3.36]
Scudamore, 2000	0	18	0	43		Not estimable
**Subtotal (95% CI)**		**491**		**302**	**66.9%**	**0.61 [0.25, 1.49]**
Total events	10		10			
Heterogeneity: Chi² = 1.01, df = 2 (P = 0.60); I² = 0%						
Test for overall effect: Z = 1.09 (P = 0.28)						
**3.4.2 not including segment I resection**						
Hu, 2003	0	52	2	63	12.7%	0.23 [0.01, 4.99]
Wu, 1999	0	15	1	25	6.3%	0.53 [0.02, 13.77]
Yang, 2014	0	350	2	346	14.2%	0.20 [0.01, 4.11]
**Subtotal (95% CI)**		**417**		**434**	**33.1%**	**0.27 [0.05, 1.62]**
Total events	0		5			
Heterogeneity: Chi² = 0.21, df = 2 (P = 0.90); I² = 0%						
Test for overall effect: Z = 1.43 (P = 0.15)						
**Total (95% CI)**		**908**		**736**	**100.0%**	**0.50 [0.23, 1.09]**
Total events	10		15			
Heterogeneity: Chi² = 1.77, df = 5 (P = 0.88); I² = 0%						
Test for overall effect: Z = 1.74 (P = 0.08)						
Test for subgroup differences: Chi² = 0.61, df = 1 (P = 0.43), I² = 0%						

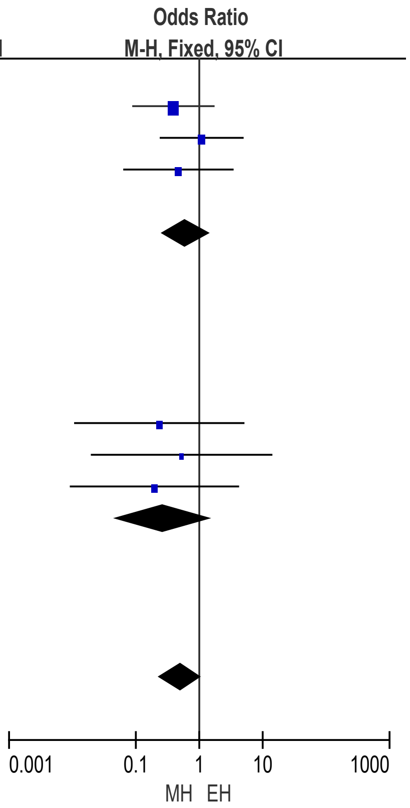

Fig G Subgroup analysis of mortality rate by type of MH

	MH			EH				Mean Difference
Study or Subgroup	Mean	SD	Total	Mean	SD	Total	Weight	IV, Random, 95% CI
**4.4.1 including segment I resection**								
Chen, 2014	244	55	118	223	40	80	19.0%	21.00 [7.76, 34.24]
Cheng, 2012	230	84	63	316	97	41	17.1%	-86.00 [-122.22, -49.78]
Qiu, 2013	259	192	292	211	162	138	17.2%	48.00 [13.14, 82.86]
Scudamore, 2000	238	89	18	304	83	43	15.6%	-66.00 [-114.02, -17.98]
**Subtotal (95% CI)**			**491**			**302**	**68.9%**	**-18.92 [-76.96, 39.13]**
Heterogeneity: Tau² = 3192.96; Chi² = 43.75, df = 3 (P < 0.00001); I² = 93%								
Test for overall effect: Z = 0.64 (P = 0.52)								
**4.4.2 not including segment I resection**								
Hu, 2003	265	77	52	264	89	77	17.8%	1.00 [-27.86, 29.86]
Wu, 1999	474	78	15	348	138	25	13.2%	126.00 [59.03, 192.97]
**Subtotal (95% CI)**			**67**			**102**	**31.1%**	**59.70 [-62.57, 181.97]**
Heterogeneity: Tau² = 7120.38; Chi² = 11.29, df = 1 (P = 0.0008); I² = 91%								
Test for overall effect: Z = 0.96 (P = 0.34)								
**Total (95% CI)**			**558**			**404**	**100.0%**	**4.11 [-39.09, 47.31]**
Heterogeneity: Tau² = 2506.66; Chi² = 55.72, df = 5 (P < 0.00001); I² = 91%								
Test for overall effect: Z = 0.19 (P = 0.85)								
Test for subgroup differences: Chi² = 1.30, df = 1 (P = 0.25), I² = 22.8%								

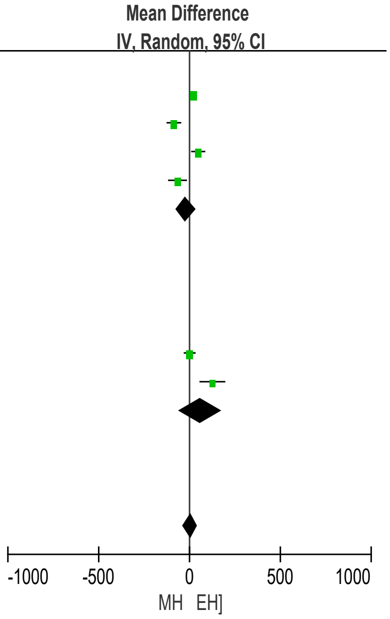

Fig H Subgroup analysis of operation time by type of MH

Study or Subgroup	log[Hazard Ratio]	SE	Weight	Hazard Ratio IV, Random, 95% CI	Year	Hazard Ratio IV, Random, 95% CI
Hu, 2003	0.08167803	0.06771345	27.9%	1.09 [0.95, 1.24]	2003	
Cheng, 2012	0.18232156	0.06574161	28.5%	1.20 [1.05, 1.37]	2012	
Yang, 2014	-0.158224	0.0026169	0.0%	0.85 [0.85, 0.86]	2014	
Chen, 2014	0	0.00887866	43.7%	1.00 [0.98, 1.02]	2014	
**Total (95% CI)**			**100.0%**	**1.08 [0.96, 1.21]**		

Heterogeneity: Tau² = 0.01; Chi² = 8.87, df = 2 (P = 0.01); I² = 77%

Test for overall effect: Z = 1.27 (P = 0.20)

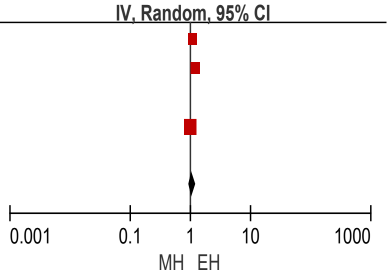

Fig I Sensitivity analysis of overall survival via removing the study manifested by high heterogeneity
